# Preclinical models of female pelvic pain disorders

**DOI:** 10.1097/j.pain.0000000000004016

**Published:** 2026-05-20

**Authors:** Irene Scuffi, Matilde Marini, Felice Petraglia, Lorenzo Landini, Romina Nassini, Francesco De Logu

**Affiliations:** a Department of Health Sciences, Clinical Pharmacology and Oncology Section, University of Florence, Florence, Italy; b Department of Experimental, Clinical and Biomedical Sciences “Mario Serio”, University of Florence, Florence, Italy; c Department of Maternal and Child Health, Careggi University Hospital, Florence, Italy

**Keywords:** Pain, Sex, Women, Endometriosis, Adenomyosis, Dysmenorrhea, Vulvodynia, Uterine leiomyomas, Pelvic inflammatory disease

## Abstract

Pain is influenced by a complex interplay of biological, psychological, and social factors. Sex has emerged as a key determinant of vulnerability to chronic pain and a major risk factor for poor response to available pharmacological treatments. Women report higher rates of chronic pain and exhibit greater pain sensitivity; however, the underlying mechanisms remain poorly understood. Preclinical models are essential to uncover sex-specific biological pathways involved in pain and to guide the development of novel, targeted therapies through translational approaches. This review summarizes animal models of female-associated pelvic pain disorders, including endometriosis, adenomyosis, dysmenorrhea, vulvodynia, interstitial cystitis, uterine leiomyomas, chronic pelvic pain, and pelvic inflammatory disease. These models recapitulate key features such as lesion biology, neuroimmune interactions, and pain behaviors also observed in patients. However, current models still face limitations in capturing spontaneous pain dynamics, hormonal complexity, and psychosocial influences. Refining and integrating biological, behavioral, and sex-specific endpoints will be crucial to enhance their translational relevance and advance precision pain therapies for women.

## 1. Introduction

Pain perception is highly subjective and influenced by multiple biological, psychological, and social factors. Pain arising from disorders of the female pelvic region represents a major yet frequently underrecognized health burden. Female pelvic pain disorders, including endometriosis, adenomyosis, dysmenorrhea, vulvodynia, uterine leiomyomas, and pelvic inflammatory disease (PID), affect millions of women worldwide and represent a major cause of chronic pain during the reproductive years. These conditions substantially impair quality of life, reproductive health, and psychological well-being, often interfering with daily and social activities and sexual function.^26,59,67,95^

Pelvic pain associated with gynecological disorders is frequently persistent or recurrent and may occur independently of the extent of visible pathology. In many patients, pain persists even after treatment of the underlying disease, suggesting that mechanisms beyond tissue damage contribute to symptom maintenance. Clinical manifestations often include visceral hypersensitivity, referred pain, dyspareunia, dysmenorrhea, and comorbid pain disorders, highlighting the complex and multifactorial nature of these conditions.^13,91^

The mechanisms underlying these pain states involve complex interactions between inflammatory mediators, immune responses, and neuroplastic changes in peripheral and central nociceptive pathways, with hormonal factors playing a key role in modulating pain perception and nociceptor sensitivity, thereby contributing to dynamic fluctuations in pain state.^34^ In addition, cognitive, affective, and behavioral components may further shape the pain experience in women.^86^

Despite their high prevalence and clinical impact, the biological mechanisms driving female pelvic pain disorders remain poorly understood, thus contributing to delayed diagnosis, limited treatment options, and suboptimal pain management. Current therapeutic strategies are frequently directed toward the underlying gynecological condition or inflammation, while effective mechanism-based treatments specifically targeting pain are still lacking.

Preclinical models play a critical role in dissecting these mechanisms and in identifying novel therapeutic targets. Experimental animal models allow controlled investigation of disease-driven processes, including lesion formation, inflammation, hormonal modulation, and neuroimmune signaling, while enabling the evaluation of pain-related behavioral outcomes.

In this review, we summarize currently available preclinical animal models of pelvic pain disorders affecting the female reproductive system, focusing on conditions such as endometriosis, adenomyosis, dysmenorrhea, vulvodynia, uterine leiomyomas, and pelvic inflammatory disease. We highlight experimental models that reproduce key disease-driven pathological processes and associated pain behaviors. For each disorder, we discuss experimental approaches used to model disease-associated pain, their advantages, and relevance for understanding the biological mechanisms underlying pelvic pain in women. By integrating findings across experimental systems, this review also aims to highlight current limitations in pain modeling and to identify priorities for future research aimed at improving the translational relevance of preclinical studies in female pelvic pain disorders.

## 2. Methods

This review was conducted through a comprehensive literature search aimed at summarizing and critically discussing preclinical studies investigating animal models of female-associated pain disorders. The objective was to provide a pain-focused perspective on experimental models used to study endometriosis, adenomyosis, dysmenorrhea, vulvodynia, uterine leiomyomas, and pelvic inflammatory disease. Relevant studies were identified through PubMed, Scopus, and Web of Science using combinations of keywords related to pain, animal models, and female pelvic disorders. No restrictions on publication year or language were applied, and only peer-reviewed articles were included.

Priority was given to preclinical in vivo studies incorporating pain-related behavioral or functional endpoints, including mechanical or thermal hypersensitivity, visceral pain responses, referred pain, or spontaneous pain-like behaviors. Studies distinguishing between evoked and ongoing pain were emphasized due to their translational relevance. As pain phenotyping remains limited in several female pelvic disorders, seminal studies focused on model establishment, inflammation, hormonal regulation, or tissue remodeling were also included when widely used in the field. Human clinical studies were not systematically reviewed but selectively cited to contextualize preclinical findings and highlight discrepancies between experimental outcomes and clinical pain phenotypes. Study selection was guided by relevance to pain neurobiology and translational potential.

## 3. Results

### 3.1. Endometriosis

Endometriosis is a gynecological condition characterized by the growth of endometrial tissue in extrauterine sites, causing severe chronic pelvic pain and infertility.^67^ Approximately 10% of women of fertility age are affected by endometriosis, and the diagnostic process is often delayed due to the lack of symptom specificity.^67^ More than 60% of women affected by endometriosis report chronic pelvic pain, with a likelihood of experiencing abdominal pain that is over 10 times higher than that observed in healthy women.^13^ Surgical removal of endometriotic lesions alleviates chronic pain in approximately 70%-80% of patients^3^; however, pain frequently recurs within 12 months after lesion excision, even in the absence of lesion regrowth.^120,132^

#### 3.1.1. Preclinical model and pain-related outcome

Murine models of endometriosis are essential for dissecting the pathogenesis of this estrogen-dependent inflammatory disease, although their development is complicated by the absence of spontaneous menstruation in mice, requiring experimental induction of lesions. Most current models rely on ovariectomy or transplantation of isolated endometrial tissue, representing the oldest and most widely used approaches and remaining consistent with key aspects of the human disease process.^58,134^ In surgical engraftment models, uterine tissue fragments are sutured or adhered to the peritoneal wall, mesentery, or abdominal surfaces, generating cyst-like, gland-containing lesions resembling those observed in patients.^32,35^ Alternative approaches based on intraperitoneal (i.p.) injections more closely reproduce retrograde menstruation by dispersing minced or decidualized endometrial tissue into the peritoneal cavity, enabling investigation of implantation dynamics and immune interactions, although with greater variability in lesion distribution and number.^50,70,135^

An ovarian endometriosis model established through bursectomy has been used to investigate ovarian function in mice.^74^ Transplantation of minced uterine tissue into the ovarian bursal space resulted in altered ovarian anatomy, cystic lesions, and increased oxidative stress markers, including 4-hydroxy-2-nonenal (4-HNE) and 8-hydroxy-2′-deoxyguanosine (8-OHdG), particularly in primordial and pre-antral follicles. Reduced follicle-stimulating hormone (FSH) levels were associated with impaired follicular development and reduced fertility.^74^ Similarly, surgical models in rats confirmed the presence of endometrial glandular tissue within the ovary and reported increased numbers of unruptured luteinized follicles, suggesting ovarian dysfunction despite unchanged body weight.^84^

Fattori et al. developed a nonsurgical murine model mimicking key features of human endometriosis.^58^ Estradiol-primed donor endometrial tissue was minced and injected intraperitoneally into recipient mice, leading to a time-dependent increase in endometriotic-like lesions and the development of both evoked and spontaneous pain behaviors. Pharmacological validation showed that clinically used drugs, including letrozole and danazol, reduced pain-like responses. Using the same model, Titiz et al.^143^ identified neuroimmune mechanisms involving Schwann cell C5a receptor (C5aR1), NLRP1/IL-1β activation, macrophage recruitment, oxidative stress, and TRPA1 channel activation sustaining neuroinflammation.

More recently, Wilson et al. (2020) developed a genetically engineered mouse model (GEMM) reproducing invasive spread of endometrial tissue by combining ARID1A loss and PIK3CAH1047R activation in endometrial epithelial cells through the lactotransferrin-Cre system. Unilateral salpingectomy enabled mutant cells to enter the peritoneal cavity, resembling retrograde menstruation. Limitations include reduced lifespan due to endometrial dysfunction and the complete penetrance of mutations, which differs from the mosaic nature of human disease.^151^

**Figure 1.**
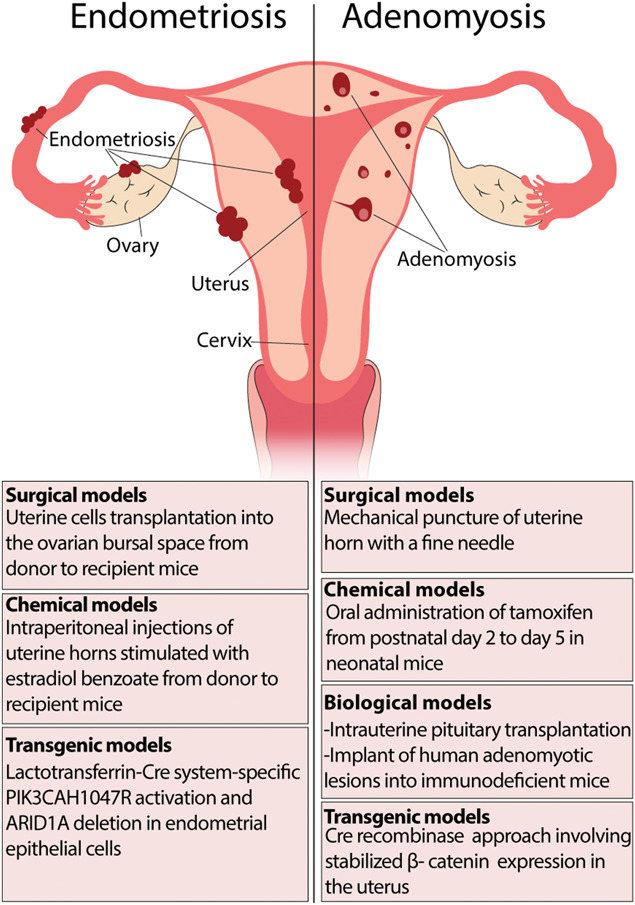
Illustration representing the differences between endometriosis and adenomyosis and related rodent models. In endometriosis, cells like the uterine lining (endometrium) grow outside the uterus within the pelvic or abdominal cavity, whereas in adenomyosis, these cells invade the muscular wall of the uterus.

Overall, while surgical and transplantation-based models provide important insights into ovarian dysfunction and fertility, they often fail to fully reproduce the complex symptomatology of endometriosis, particularly spontaneous pain and systemic inflammatory alterations beyond local lesions. Nonsurgical approaches, such as those developed by Fattori et al. and further characterized by Titiz et al. represent an important advancement in reproducing the human pain phenotype and dissecting neuroimmune mechanisms underlying disease-associated pain^58,143^ (Fig. 1).

Rodent models offer practical advantages, including low cost, ease of handling, and genetic manipulability, but their translational value for endometriosis-associated pain is limited by the absence of menstruation. Nonhuman primate models more closely recapitulate key reproductive and hormonal features of the human condition and can develop spontaneous endometriosis with lesions similar to those observed in women.^5,36,46,100^ The spontaneous incidence of endometriosis is relatively high, approximately 36% in rhesus monkeys, 27% in baboons, and 29% in cynomolgus monkeys,^4,37,47^ making these species particularly suitable for investigating the natural history and pathogenesis of the disease.

These models have provided important insights into inflammatory and immune processes, including macrophage activation and cytokine release, that are directly relevant to nociceptor sensitization and pain generation.^8,9^ Moreover, primate studies have supported the evaluation of anti-inflammatory strategies with potential analgesic implications, such as tumor necrosis factor-α inhibition.^16,38^ Despite their cost and ethical constraints, nonhuman primate models remain valuable for understanding pain-relevant mechanisms that are difficult to capture in rodent systems.

In summary, each model has intrinsic limitations, underscoring the need for integrative strategies that combine functional, histological, and behavioral endpoints to better mirror the multifaceted nature of endometriosis in women.

### 3.2. Adenomyosis

Adenomyosis is a status characterized by the infiltration of endometrial tissue into the myometrium and is often described as a disorder closely related to endometriosis.^49^ Importantly, adenomyosis and endometriosis may co-exist in the same patient, further complicating diagnosis and management.^161^ Similar to endometriosis, adenomyosis is frequently associated with chronic pelvic pain, infertility, and dysmenorrhea.^25^

Clinical studies indicate that approximately 70% of patients with adenomyosis report symptoms related to pain. Regarding dysmenorrhea, patients are almost evenly distributed across mild, moderate, and severe pain categories. In addition, nearly 25% of women with adenomyosis experience chronic pelvic pain, often accompanied by dyspareunia and anorectal pain.^27^ Nonsteroidal anti-inflammatory drugs^2^ and the levonorgestrel-releasing intrauterine system^102^ are currently among the most used therapeutic options for the management of pain associated with adenomyosis. However, despite the high clinical burden of pain in this condition, no standardized or evidence-based clinical guidelines are currently available for the treatment of pain associated with adenomyosis.

#### 3.2.1. Murine models

Little is known about the pathogenesis of adenomyosis, a knowledge gap that complicates its diagnosis. Several experimental strategies have been employed to model adenomyosis in mice, including pituitary engraftment, neonatal tamoxifen administration, and xenotransplantation of human tissues. In addition, GEMMs have been established to better reproduce the disease similar to that in humans.^101^

Early mouse models exploited the high spontaneous incidence of adenomyosis in specific inbred strains (eg, SHN, SLN, CD-1, SMXA, GR/A, and C3H/He). Suppression of pituitary function reduced disease development, whereas intrauterine pituitary transplantation induced adenomyosis, establishing the pituitary engraftment model as a widely used experimental approach.^71,105,108^

More recently, Zhu et al. (2023) developed a neonatal mouse model in which adenomyosis was induced through oral tamoxifen administration from postnatal day 2 to day 5.^164^ Treated mice exhibited weight gain and increased sensitivity in the hot plate test, indicative of hyperalgesia. Resveratrol treatment improved generalized hyperalgesia and modulated biomarkers implicated in adenomyosis, including HMGB1, IL-33, osteopontin, PCNA, p-p65, RAGE, and TLR4. It also increased GAD65-expressing neurons in the nucleus raphe magnus, suggesting enhanced GABAergic inhibitory control, and reduced TRPV1 activity, previously correlated with disease severity.^109^

Several studies have employed xenotransplantation approaches, implanting human adenomyotic lesions into immunodeficient mice, allowing monitoring of lesion establishment, progression, and therapeutic responses in a controlled in vivo environment.^28,76,141,163^

A surgical puncture model has also been described, in which mechanical injury to one uterine horn induces adenomyosis while the contralateral horn serves as control.^64^ Lesion volume increased over time, accompanied by enhanced stromal proliferation, vascularization, and fibrosis. Affected mice showed earlier delivery and reduced litter size despite comparable implantation numbers.^52^

Although limited, studies using GEMMs have highlighted molecular mechanisms underlying adenomyosis, particularly aberrant β-catenin signaling. Increased β-catenin activation disrupts uterine morphogenesis, cellular differentiation, and tissue remodeling, promoting ectopic endometrial growth within the myometrium.^110,142^ These findings suggest that dysregulation of developmental pathways contributes to disease pathogenesis and may identify novel therapeutic targets (Fig. 1).

Overall, current experimental models have advanced understanding of uterine remodeling, lesion progression, and pain-related mechanisms in adenomyosis, although important limitations remain. Neonatal tamoxifen-induced models are valuable for mechanistic studies, whereas surgical puncture models allow longitudinal evaluation and assessment of reproductive outcomes. However, rodent models are limited by fundamental reproductive differences that restrict translational interpretation and prevent investigation of hallmark symptoms such as menorrhagia and dysmenorrhea. Hormone-induced models may also introduce systemic confounders. Despite these constraints, rodent studies provide valuable evidence paralleling human pathology, particularly in relation to impaired fertility.

#### 3.2.2. Nonhuman primates and other species models

Spontaneous adenomyosis has been documented primarily in nonhuman primates, particularly rhesus macaques and baboons, with additional naturally occurring cases reported in several nonprimate species.^101^ These models offer the advantage of studying disease development in a physiological hormonal context, especially in species whose reproductive anatomy and endocrine regulation closely resemble those of humans. In baboons and rhesus macaques, spontaneous adenomyosis has been described mainly through case reports and has been associated with uterine remodeling and inflammation, even in the absence of coexisting endometriosis.^15–17^ Such processes are highly relevant to nociceptor activation and chronic pain generation. Beyond primates, reports of spontaneous adenomyosis in other species, including horses, dogs, cats, rabbits, rats, and guinea pigs, remain sparse and largely descriptive.^12,60,64,103,138^ The limited assessment of pain-related behaviors in these models likely reflects a lack of systematic sensory phenotyping rather than absence of nociceptive relevance. Overall, the scarcity of well-characterized spontaneous models represents a significant missing point in translational pain research and underscores the need for improved integration of pain endpoints into studies of adenomyosis.

Overall, research on pain associated with adenomyosis remains underdeveloped, mainly focused on generalized hyperalgesia rather than clinically relevant pelvic pain. Although up to one-third of women with adenomyosis remain asymptomatic, severe and persistent chronic pelvic pain is frequently reported, with evidence supporting a correlation between the extent of myometrial infiltration and pain severity.^127,128^ Further refinement and integration of preclinical models are therefore required to improve their translational value and ensure closer alignment between experimental findings and clinical management.

### 3.3. Dysmenorrhea

Dysmenorrhea is the most common gynecological condition that refers to painful menstrual cramps occurring either before or during menstruation. It primarily affects adolescents and young women, significantly impacting the health and daily lives of millions worldwide, with a reported prevalence ranging from 34% to 94%^59,77^ and 2% to 29% of women experiencing severe pain.^104^ There are 2 types of dysmenorrhea: primary dysmenorrhea (PD) (not associated with other diseases) and secondary dysmenorrhea (SD) (associated with conditions like endometriosis).^121,144^ Importantly, PD may be of specific interest to preclinical and clinical research, given the mounting evidence that it may be a risk factor for the later development of other conditions associated with pain, both gynecological (ie, endometriosis,^31^) or associated with pain sensitivity.^77^ Nonsteroidal anti-inflammatory drugs are considered the first-line treatment for PD, although their use is frequently limited by adverse effects, including headache and gastrointestinal toxicity.^89^ Paracetamol (acetaminophen) is also used as an analgesic option; however, its predominant central nervous system activity and relatively weak peripheral anti-inflammatory effects reduce efficacy, making it a second-line treatment.^44^ Hormonal therapies, including hormonal contraception, represent an effective option, particularly for women not seeking pregnancy. Estrogens suppress FSH release and prevent ovulation, while progesterone reduces endometrial thickness and cervical mucosal proliferation, decreasing arachidonic acid availability for prostaglandin synthesis and thereby reducing uterine contractions during menstruation.^131^ Gonadotropin-releasing hormone (GnRH) agonists represent an additional therapeutic option, inhibiting LH and FSH secretion and suppressing estrogen production; these agents are generally reserved for cases refractory to nonsteroidal anti-inflammatory drugs and hormonal therapies.^1^

#### 3.3.1. Mechanistic basis and rodent models of primary dysmenorrhea

**Figure 2.**
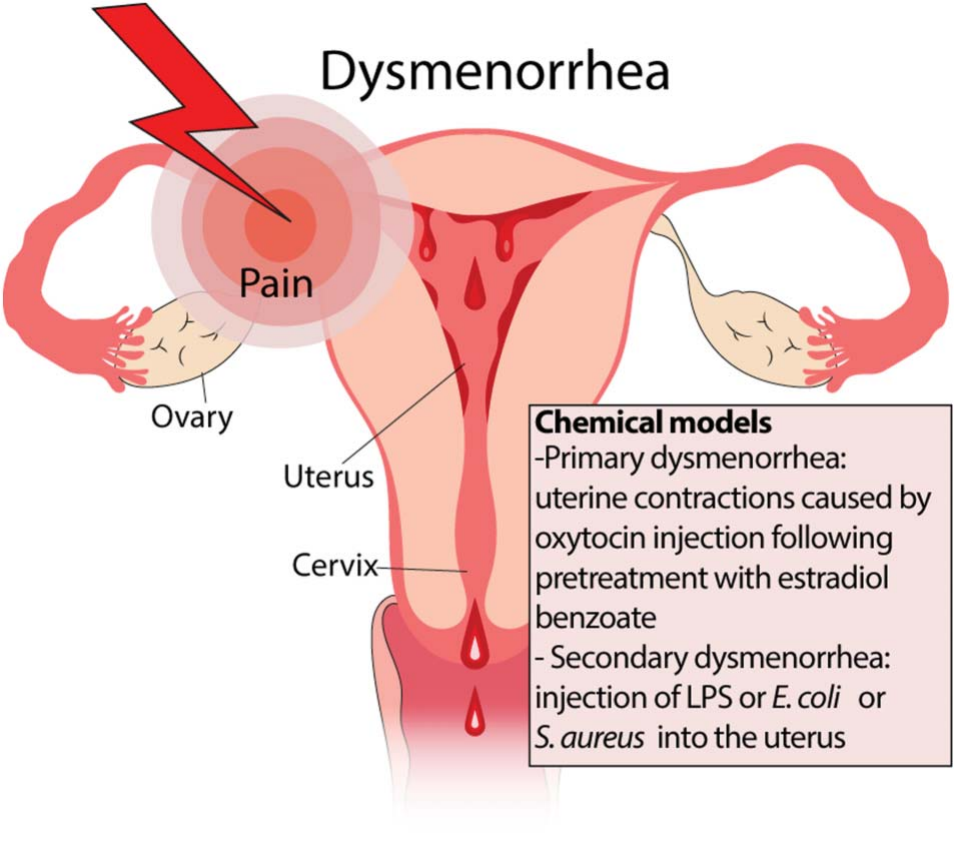
Illustration representing the pain associated with menstrual bleeding (dysmenorrhea) and related rodent models. LPS, lipopolysaccharide.

Owing to their short and regular estrous cycles, rodents are the most used models for studying dysmenorrhea. Primary dysmenorrhea is often accompanied by additional symptoms, including lower abdominal pain, sweating, and fatigue.^83^ Much evidence indicates that excessive PG production during menstruation is the main driver of PD.^24^ PGF2α induces vasospasm and hypercontractility of uterine smooth muscle, leading to uterine ischemia, hypoxia, and ultimately lower abdominal cramps. To investigate PD, researchers commonly employ in vivo models based on oxytocin-induced uterine contractions, particularly in mice. In uterine smooth muscle cells, oxytocin binds to oxytocin receptors (OTRs), enhancing contractile activity.^150^ Oxytocin also induces the release of PGF2α from endometrial cells, which activates the PG receptor and increases the synthesis of OTR within the uterus.^156^ All these cellular events lead to an increase in intracellular calcium [Ca^2+^]_i_, which triggers excessive uterine contractions and underlies the pathophysiology of dysmenorrhea.^88,118^ The general experimental procedure described by several authors involves monitoring the estrous cycle of nonpregnant female mice of different strains^80,157^ for approximately 16 days (4 cycles). Mice are then pretreated (i.p.) with estradiol benzoate for at least 3 consecutive days to sensitize the uterus, since estrogen promotes OTR overexpression and increases uterine responsiveness to oxytocin.^144,157^ Subsequently, oxytocin is administered (i.p.) to induce abdominal contractions, commonly referred to as writhing, which is associated with pain behaviors.^29,157^ These behaviors typically consist of pelvic rotation followed by hind limb stretching and abdominal wall contractions. Pain intensity was scored from 0 to 3, where 0 refers to normal body position and behaviors; 1 refers to body leaning to the left or right; 2 refers to stretching of the hind limbs and dorsiflexion of the hind paws, with the body stretched and flat on the bottom and the pelvis rotated sideward; and 3 refers to abdominal muscle contraction followed by body stretching and hind limb extension.^130,157^ This animal model reproduces several features of human PD, including reduced myometrial area, diminished arterial blood flow, increased OTR expression, and elevated PGF2α levels, and is widely applied to evaluate the analgesic effects of drugs and herbal medicines.^112^ Moreover, it offers advantages such as simplicity, cost-effectiveness, and avoidance of surgical trauma or infection risks^153^ (Fig. 2).

#### 3.3.2. Secondary dysmenorrhea models

Owing to the complexity and incomplete understanding of its chemical process, SD has a far smaller animal model than PD. Three prevalent primary diseases of SD are known to exist: endometriosis, adenomyosis (whose models have been previously described) and endometritis.^144^ Secondary dysmenorrhea induced by endometritis is generally obtained by the injection of lipopolysaccharide into the uterine cavity.^14,162^ Another approach to induce endometritis is the injection of bacteria, such as *E. coli*^97^ or *Staphylococcus aureus*^63,159^ into the uterus. Murine uteri are generally collected 24 hours after endometritis induction for further analysis (Fig. 2).

Overall, while animal models of dysmenorrhea, particularly those mimicking PD, have provided important insights into the underlying mechanisms and facilitated the evaluation of novel therapies, modeling SD remains more challenging due to its multifactorial and disease-specific etiology. In both PD and SD models, pain is typically assessed through behavioral endpoints such as writhing responses, abdominal contractions, and altered locomotor activity, which reflect visceral pain states and allow a comprehensive evaluation of dysmenorrhea-associated pain. Nevertheless, the development of refined and standardized models that better recapitulate the complexity of human SD and integrate multidimensional pain assessment is crucial. Such advancements will not only deepen our understanding of the pathophysiology of dysmenorrhea but also accelerate the discovery of more effective and targeted treatments to improve the quality of life for affected women.

### 3.4. Vulvodynia

Vulvodynia is a complex condition characterized by chronic pain or discomfort in the vulva, lasting at least 3 months without underlying identifiable causes. It is currently classified according to pain distribution as either generalized, involving the entire vulva (generalized vulvodynia), or localized, affecting specific regions such as the clitoris (clitorodynia) or the vaginal vestibule (vestibulodynia).^126^ It can be present during sexual or nonsexual situations and occurs in around 10% of women of all ages.^18^ The etiology of vulvodynia has not yet been fully elucidated. Proposed contributing factors include alterations in nociceptive signaling due to nerve damage or irritation along the vulva-spinal cord axis, an increased density and heightened excitability of vulvar sensory fibers, local inflammatory processes characterized by elevated cytokine production, atypical sensitivity to external stimuli, inherited susceptibility, and dysfunction of the pelvic floor musculature, such as impaired tone, involuntary contractions, or instability.^20,55,145^ Management of vulvodynia requires a multidisciplinary, patient-centered approach that prioritizes recognition and validation of the patient's pain. Treatment typically integrates vulvar self-care strategies, pharmacological neuromodulation with low-dose oral agents (such as tricyclic antidepressants, serotonin and norepinephrine reuptake inhibitors, and anticonvulsants), and topical compounded therapies applied directly to the vulva, provided they are free of allergens.^87,122,136^ Pelvic floor focused on women's health physical therapy represents a key component of care, addressing muscle weakness, spasm, and dysfunction through targeted exercises and manual techniques. Adjunctive interventions, including nerve blocks, psychological and mindfulness-based therapies, neurostimulation, and, in selected refractory cases of vulvodynia, surgical approaches, may further contribute to symptom control and functional recovery.^72,75^

#### 3.4.1. Inflammatory and infection-driven models of vulvodynia

The first preclinical models of vulvodynia were established by Farmer et al. in 2011,^57^ demonstrating that vulvar mechanical allodynia could be induced in mice through repeated infection with *Chlamydia albicans* or localized injection of zymosan, a yeast cell wall component that triggers inflammation. These complementary paradigms, infection-based and inflammation-based, produced durable vulvar hypersensitivity that persisted after resolution of the initial trigger, closely resembling provoked vulvodynia in women. Affected mice also displayed vulvar hyperinnervation, a hallmark feature observed in patients, strengthening the translational relevance of the model. This work provided the first experimental evidence that infection- or inflammation-induced peripheral changes can lead to persistent vulvar pain, establishing a robust platform for mechanistic and therapeutic studies.^57^

Subsequent studies using repeated vulvar zymosan administration further investigated inflammatory mechanisms, highlighting the role of the nerve growth factor (NGF) pathway.^10^ Nerve growth factor release following mast cell accumulation, together with pro-inflammatory cytokines and neuropeptides such as IL-1β, CGRP, and IL-6, was associated with increased transcription of TRP channels (TRPV1, TRPA1) and sodium (Na^+^) channels. Pharmacological blockade of NGF signaling prevented vulvar pain development and reduced TRPV1 and TRPA1 expression, supporting NGF as a key mediator of inflammation-driven vulvar allodynia and a potential therapeutic target.

Another model employed female mice injected with complete Freund's adjuvant (CFA) at the distal level of the uterus.^133^ Although behavioral changes were limited, CFA induced local inflammation, macrophage infiltration, vascular proliferation, and vaginal hyperinnervation. Castro et al.^22^ demonstrated that clodronate-mediated macrophage depletion prevented vestibular hypersensitivity, while Chakrabarty et al.^23^ showed that CFA injection in rats increased vestibular mechanical sensitivity and that blockade of angiotensin II receptor type 2 (AT2R) or inhibition of renin-angiotensin system (RAS) proteases prevented sensory nerve growth and hyperinnervation, indicating a key role for local inflammatory RAS signaling.

**Figure 3.**
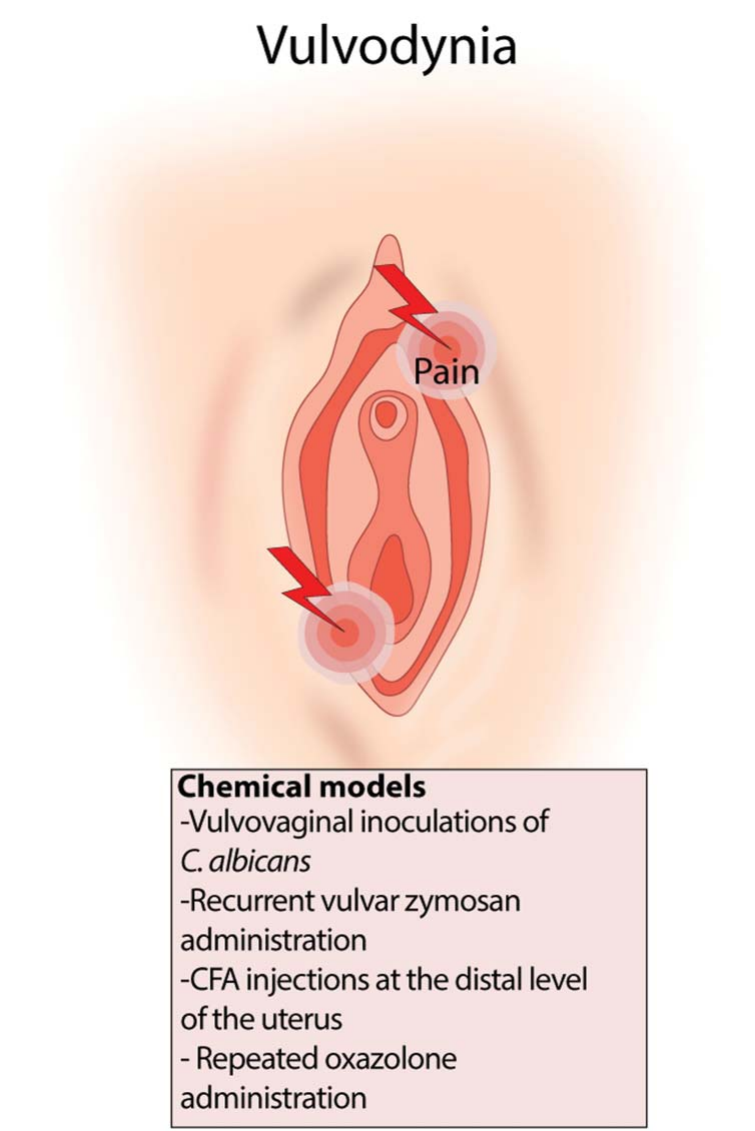
Illustration representing vulvodynia-related vulvar pain in women and related rodent models. CFA, complete Freund's adjuvant.

Histopathological observations have also guided model development. Vestibular biopsies from patients consistently show mast cell accumulation and hyperinnervation.^19,69^ Based on these findings, Landry et al. (2017) developed an oxazolone-induced model in mice that reproduced increased vestibular innervation and mast cell infiltration. This inflammatory environment increased histamine levels and upregulated Ngf and Cadm1 expression, suggesting early molecular changes driving aberrant innervation and mechanical hypersensitivity^93^ (Fig. 3).

Collectively, these models have provided important insights into vulvodynia pathophysiology, emphasizing the roles of infection, inflammation, mast cell activation, neuro-immune signaling, and aberrant innervation in persistent vulvar pain. Although each model captures distinct aspects of the disorder, they consistently highlight immune-neuronal interactions and growth factor signaling, particularly NGF, as central mechanisms driving chronic pain. These experimental platforms also identify potential therapeutic targets, including mast cells, NGF, TRP channels, and the RAS, supporting the development of more effective treatment strategies for vulvodynia.

### 3.5. Uterine leiomyomas

Uterine leiomyomas, commonly referred to as myomas or fibroids, are the most frequent pelvic tumors in women, affecting 70%-80% during reproductive years.^11,68^ Although up to 70% of fibroids are asymptomatic and require neither treatment nor routine follow-up,^147^ a substantial proportion of patients develop clinically significant symptoms. Depending on size, number, and location, symptomatic leiomyomas may cause pelvic pressure, abdominal or back pain, bloating, abnormal uterine bleeding, dysmenorrhea, urinary and bowel dysfunction, sexual dysfunction, infertility, and adverse obstetric outcomes such as preterm labor, malpresentation, and fetal growth restriction. Pain represents one of the most prevalent and disabling manifestations, including chronic pelvic pain and pain related to mass effect or inflammation, and significantly contributes to emotional distress, depression, and reduced quality of life.^66,137^ Despite its clinical relevance, the biological mechanisms underlying fibroid-associated pain remain poorly defined, and pain relief is often incomplete with current treatments. Management strategies include surgical, radiological, and medical approaches primarily aimed at reducing tumor volume and controlling bleeding, with pain relief often considered secondary. Minimally invasive procedures such as laparoscopic cryomyolysis and thermocoagulation reduce fibroid volume by disrupting blood supply through thermal injury.^65^ Among nonsurgical options, uterine artery embolization induces fibroid ischemia and degeneration, improving bleeding and pressure-related symptoms,^158^ although it is associated with higher rates of minor complications, re-intervention, and uncertain effects on fertility. MRI-guided focused ultrasound surgery (MRgFUS) offers a noninvasive alternative allowing precise thermal ablation under imaging guidance, but its use is limited by strict eligibility criteria, high costs, and retreatment rates.^119^ Medical therapies are mainly adjunctive or temporary. GnRH agonists reduce fibroid size and vascularization but are limited by adverse effects with long-term use.^45^ Tranexamic acid may reduce menstrual bleeding but shows inconsistent benefit in fibroid-associated menorrhagia,^117^ while selective estrogen receptor modulators and vitamin D supplementation show potential biological effects despite limited clinical evidence.^117^ Overall, management should be individualized according to symptom severity, particularly pain, reproductive plans, and long-term risks. However, the absence of therapies specifically targeting fibroid-associated pain highlights an important unmet clinical need.

#### 3.5.1. Preclinical models of fibroid development and growth

Despite the high prevalence and clinical impact of uterine fibroids, their etiology and pathophysiology remain incompletely understood, partly due to the limited availability of reliable in vivo models. Over the past 3 decades, several rodent models have been developed to investigate fibroid biology, some providing insight into mechanisms potentially underlying fibroid-associated pain. Chemically induced models in mice rely on repeated administration of estradiol benzoate combined with progesterone, promoting myometrial hyperplasia, extracellular matrix deposition, and fibroid-like lesions characterized by increased uterine thickness and collagen content.^48^ These models are inexpensive and reproducible, but they lack of the genetic and molecular alterations that drive human leiomyomas.

Biologically induced models are based on xenotransplantation of human leiomyoma tissue or cells into immunodeficient mice. Hassan et al. (2008) established a xenograft model in SCID mice through subcutaneous implantation of human fibroid fragments with estrogen supplementation, demonstrating improved graft survival associated with COX-2 and VEGF overexpression.^73^ The grafts retained proliferation, apoptosis indices, smooth muscle markers, and steroid receptor expression comparable to human tissue. Fritsch et al. (2015) further refined this model by optimizing estradiol and progesterone supplementation in ovariectomized SCID mice, generating xenografts that preserved histological and molecular features and remained responsive to medical therapies Ishikawa et al. (2010) demonstrated that progesterone, rather than estrogen alone, drives leiomyoma growth,^78^ a finding later confirmed by Drosch et al. (2013), who showed that uncultured primary human fibroid cells could generate orthotopic tumors following intrauterine injection in hormone-supplemented SCID/beige mice.^51^ This approach produced stable xenografts closely resembling primary human leiomyomas and may better reflect disease pathogenesis.

More recently, Suzuki et al. (2018) introduced a simplified xenograft model in BALB/c nude mice, where subcutaneous implantation of human fibroid tissue with estrogen/progesterone pellets eliminated the need for Matrigel or specialized housing.^140^ Although xenograft models preserve many human features and are valuable for therapeutic testing, they depend on surgical specimens, produce relatively small grafts, and lack interaction with the uterine microenvironment.

**Figure 4.**
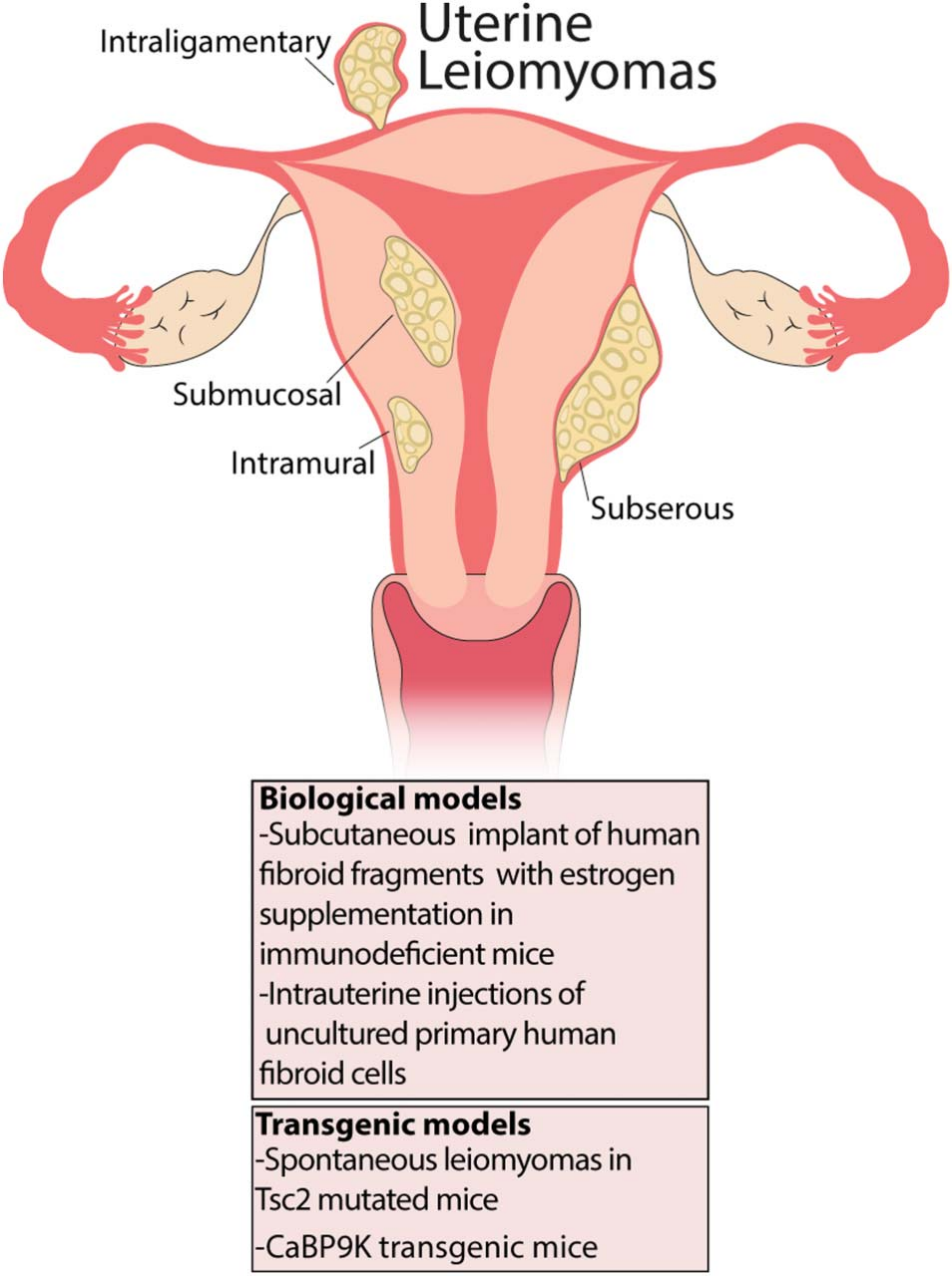
Illustration of uterine leiomyomas (fibroids) showing their typical locations within the uterus, including submucosal, intramural, subserous, and intraligamentary sites. Related rodent models are also reported.

Genetic models include the Eker rat, carrying a germline mutation in the Tsc2 gene and spontaneously developing leiomyomas in 40%-70% of female rats by 14 months.^54^ These tumors share molecular and receptor profiles with human fibroids, although atypical epithelioid tumors may also occur. Another model is the CaBP9K transgenic mouse developed by Romagnolo et al. (1996), in which SV40 Tag expression under an estrogen-responsive promoter induces estrogen-dependent leiomyomas with high penetrance.^123^ Tumor growth ceases after ovariectomy or estrogen withdrawal, although unrelated conditions such as lung adenocarcinoma and polycystic kidney disease limit lifespan (Fig. 4).

Overall, chemically induced models are inexpensive and useful for drug testing, xenograft models closely mimic human fibroid biology and are valuable for translational research, and genetic models provide mechanistic insight into hormone-driven tumorigenesis, although each system presents limitations that prevent complete replication of the human condition. Importantly, these models also provide opportunities to explore the mechanisms of pain associated with fibroid, a poorly understood but clinically relevant symptom domain.

Nonrodent models of uterine leiomyoma include species such as guinea pigs, potbellied pigs, and aging hens, each offering unique advantages for translational research. Guinea pigs spontaneously develop leiomyomas in up to 8% of female guinea pigs by 4 years of age, and continuous estrogen exposure induces tumors resembling human fibroids histologically and immunohistochemically.^61^ Potbellied pigs also show a high incidence of spontaneous uterine fibroids, with similar gross and microscopic features to those in women, making them particularly relevant due to comparable hormonal cycles and tumor biology.^107^ Leiomyomas of the ventral ligament of the oviduct occur spontaneously in domestic fowl, with a prevalence ranging from 0% to 60%, depending on the line and breed.^7^ Similar to human leiomyomas, chicken fibroids exhibit increased expression of B-cell lymphoma 2 as well as estrogen and progesterone receptors.^99^ In addition, spontaneously occurring uterine leiomyomata have been documented in several primate species, including Old World monkeys, New World monkeys, apes, and prosimians.^30,33,85,98,139^ Together, these models complement rodent systems by providing larger anatomical scale, hormonal cyclicity, and spontaneous tumor development, making them an excellent complementary model for investigating the pathophysiology of human uterine leiomyomas.

### 3.6. Pelvic inflammatory disease

The female upper reproductive tract (comprising the endometrium, fallopian tubes, ovaries, and pelvic peritoneum) is frequently affected by infections that may trigger inflammation and lead to PID.^82^ Current theoretical frameworks suggest that pathogens can ascend from the cervix or vagina to the endometrium, fallopian tubes, or adjacent structures, causing chronic inflammation, tissue injury, pelvic pain, adhesions, and fertility complications, including increased risk of ectopic pregnancy and infertility.^90^ Pelvic inflammatory disease may result from repeated acute or subclinical insults to the adnexa or from untreated infections, most often secondary to *Chlamydia trachomatis* or *Neisseria gonorrhoeae*. The diagnostic process is complicated by the frequent absence of specific symptoms, since PID may present with subtle or even asymptomatic courses. When symptomatic, it typically manifests as dyspareunia, dysuria, abnormal vaginal discharge or bleeding, and pelvic or lower abdominal pain, a constellation of nonspecific features that makes diagnosis challenging.^53^

As definitive diagnostic criteria for PID are often lacking, a low threshold for empiric treatment is recommended to prevent complications. Management should be guided by disease severity, local antimicrobial resistance patterns, and drug availability.^160^ Mild to moderate PID can generally be managed in the outpatient setting with oral antibiotic therapy, while severe cases require inpatient treatment. Recommended outpatient regimens typically include a single dose of ceftriaxone (i.m.) combined with oral doxycycline and metronidazole, or alternative fluoroquinolone-based regimens where appropriate.^129^ Inpatient management commonly involves intravenous ceftriaxone with doxycycline or a combination of clindamycin and gentamicin, followed by oral therapy to complete treatment. Alternative regimens may be used when first-line options are unavailable, although supporting evidence is less robust. All therapeutic approaches should provide coverage against *N. gonorrhoeae*, *C. trachomatis*, and anaerobic bacteria. Metronidazole is included to enhance anaerobic coverage, particularly in severe disease, but may be omitted in mild cases if poorly tolerated. In women with confirmed *Mycoplasma genitalium* infection, moxifloxacin is the treatment of choice.^124^

#### 3.6.1. Infection- and inflammation-based models of pelvic inflammatory disease

**Figure 5.**
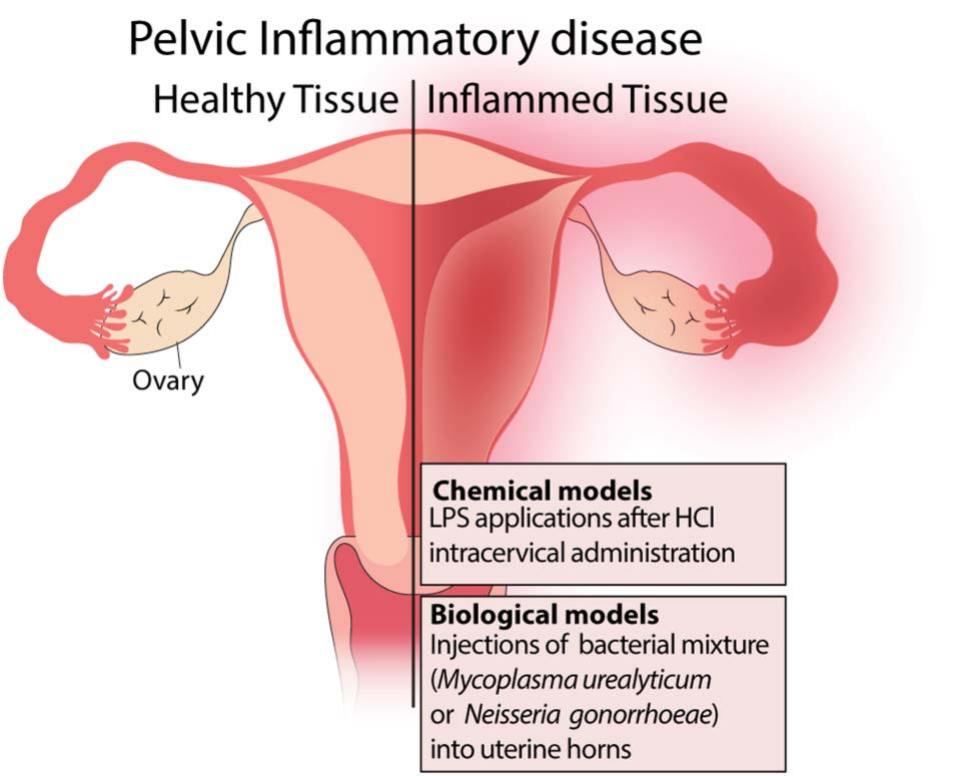
Illustration of pelvic inflammatory diseases and related rodent models are also reported. LPS, lipopolysaccharide; HCl, hydrochloric acid.

Rodent models of PID generally follow 2 strategies: (1) direct introduction of bacteria into the reproductive tract, or (2) induction of inflammatory responses that mimic PID-associated pathology. Fan et al. developed an acute PID model by mechanically exposing the rat endometrium with a needle and injecting a mixture of bacteria (eg, *E. coli*, *S. aureus*) into both uterine horns.^56^ Building on this approach, Wei et al. introduced *Mycoplasma urealyticum* in combination with these bacteria, further recapitulating polymicrobial infection.^149^ To better mimic chronic infections, Islam et al.^79^ established a murine model in which *N gonorrhoeae* (strain MS11) was inoculated transcervically into the uterine horns, allowing infection for 6 to 18 hours. In addition, nonsurgical approaches have been proposed: Oh et al. demonstrated that a single intracervical administration of hydrochloric acid (HCl) followed by 4 applications of lipopolysaccharide in female mice was sufficient to induce PID without causing acute systemic toxicity. Inflammation in this model was confirmed by elevated uterine expression of pro-inflammatory cytokines, including IL-1β, IL-6, and tumor necrosis factor-α^111^ (Fig. 5).

Pelvic inflammatory disease in nonhuman primates has been extensively used as a translational model to study human reproductive tract infections, particularly those caused by *C trachomatis*. Experimental inoculation in nonhuman primates reproduces hallmark features of human PID, including cervicitis, endometritis, salpingitis, and tubal scarring, and has been instrumental in fulfilling Koch's postulates for *C trachomatis*-induced PID.^114^ Among nonhuman primates, the pigtailed macaque and the olive baboon are especially valuable due to their reproductive tract anatomy and menstrual physiology, which closely mirror those of women. These models have provided crucial insights into the mechanisms of ascending infection,^116^ the host immune response,^146^ and the development of protective immunity.^152^ Moreover, they have been used to evaluate antibiotic efficacy^115^ and to test preventive interventions such as topical microbicides and vaccines.^113^

Altogether, these animal models provide complementary approaches to investigate how infection-driven inflammation leads to chronic tissue damage, infertility, and pelvic pain. By recapitulating key features of human disease, including inflammatory cytokine release and nerve sensitization, they represent valuable platforms to dissect the mechanisms underlying pain generation in PID and to evaluate novel preventive and therapeutic strategies.

## 4. Discussion

Chronic pain affects nearly 20% of the adult population and represents a major unmet medical need.^148^ Women are disproportionately affected, with higher prevalence and severity compared with men, particularly in female-specific disorders such as those reviewed in this study.^96^ Despite extensive investigation, the biological bases underlying sex-related differences in pain perception and persistence remain not completely understood and continue to challenge both preclinical and clinical research. The conditions discussed in this review, ranging from endometriosis, adenomyosis, dysmenorrhea, vulvodynia, uterine leiomyomas, and pelvic inflammatory disease, are not only highly prevalent but also exert profound and long-lasting effects on reproductive health, psychological well-being, and overall quality of life. Importantly, current diagnostic tools and therapeutic strategies often fail to adequately address pain associated with these diseases, underscoring a critical need for mechanism-based approaches tailored to women.

Animal models have been used for dissecting the mechanisms underlying female-specific pelvic pain. They have been implemented in several experimental setups to study pain conditions, such as neuropathic pain, cancer-related pain, migraine, and inflammatory pain.^39–43,92^ Thus, they provide essential platforms for investigating neuroimmune and hormonal pathways, validating potential therapeutic targets.

The animal models reviewed in this article reveal a striking convergence of mechanisms across clinically distinct pelvic disorders. Independent of the initiating pathology, most models engage shared biological processes that shape pain persistence and severity, providing a unifying framework to explain the overlapping and heterogeneous pain phenotypes observed in patients.

Among these convergent mechanisms, peripheral nociceptor sensitization emerges as a central driver of pelvic pain. Inflammatory mediators such as PGs, growth factors, and complement components released within diseased pelvic tissues enhance the excitability of sensory afferents innervating the viscera and surrounding structures, frequently through modulation of ion channels including TRPV1, TRPA1, and voltage-gated Na^+^ channels. These findings closely mirror clinical observations in which pain severity often poorly correlates with lesion burden or structural pathology.^6,143,155^

Neuroimmune crosstalk, involving macrophages, mast cells, and lymphocytes interacting bidirectionally with sensory neurons and glial cells, represents another shared feature across diseases. Several models demonstrate that immune-driven sensitization can persist beyond resolution of the initial insult, providing a mechanistic explanation for chronic pain states that outlast active inflammation or surgical lesion removal.^10,58,143^ This neuroimmune amplification offers insight into why pelvic pain frequently becomes chronic and treatment resistant.

Hormonal modulation further contributes to the complexity of female pelvic pain, as estrogens and progesterone influence immune responses, nociceptor excitability, and synaptic plasticity, dynamically shaping pain vulnerability across the reproductive lifespan. Experimental manipulation of hormonal status markedly alters pain outcomes, reflecting clinical observations of cyclical pain fluctuations and increased susceptibility during specific hormonal states.^34,48,131^

Finally, maladaptive peripheral plasticity, including altered function of peripheral glial cells such as Schwann cells, consistently emerges as key drivers of pain across models.^143^ These changes promote long-lasting alterations in sensory processing, lowering pain thresholds and facilitating referred and widespread pain, features commonly reported by patients with pelvic pain disorders and frequently accompanied by comorbid nonpelvic pain conditions.

Together, these convergent mechanisms provide a biological framework explaining hallmark clinical features of women's pelvic pain disorders, including heterogeneous pain phenotypes, poor correlation between lesion burden and pain severity, and frequent comorbidity with nonpelvic pain conditions. By systematically summarizing rodent models for each condition (Table 1), this review highlights both strengths and limitations, particularly regarding pain modelling and measurement. Although current models effectively reproduce lesion biology and inflammation, pain phenotyping remains heterogeneous and often incomplete. Most experimental models described in this review reproduce peripheral pelvic pathology, including inflammation, tissue remodeling, and neuroimmune interactions that initiate pain in female pelvic disorders. However, central sensitization also plays a key role in chronic pain persistence, involving altered brain sensory processing, impaired descending inhibition, and enhanced facilitatory pathways.^81,94,154^ Neuroimaging and clinical studies show structural and functional changes in pain-related regions such as the insula, thalamus, and periaqueductal gray in women with chronic pelvic pain, including endometriosis and dysmenorrhea.^21,106,125^ Importantly, these central alterations may persist even after resolution of peripheral pathology, contributing to the mismatch between disease burden and pain severity. Integrating peripheral models with approaches addressing central pain processing will therefore be essential to better understand pelvic pain mechanisms in women. Future studies should move beyond single evoked hypersensitivity measures and incorporate multidimensional assessments encompassing sensory-discriminative, affective-motivational, and functional domains, aligning more closely with clinical pain phenotyping. Refinement and standardization of outcomes capturing spontaneous pain, referred pain, and long-term sensory plasticity will be essential to improve translational relevance. Ultimately, advancing preclinical research on women's pain disorders through integrative, pain-centered experimental strategies will be crucial to improve mechanistic understanding and guide the development of more effective diagnostic tools and therapies, with the goal of enhancing women's health and quality of life.

**Table 1:** Common rodent preclinical models for the study of painful female disorders.

Disease	Model	Sex/strain	Pain endpoints/readouts
Endometriosis^74^	Surgical Uterine cells transplantation into the ovarian bursal space from donor to recipient from donor to recipient mice	♀C57BL/6N1 mice	Not evaluated
Endometriosis^84^	Surgical Ovarian endometrioma; i.p. endometriosis; extraperitoneal endometriosis	♀Sprague-Dawley rats	Not evaluated
Endometriosis^58,143^	Chemical i.p. injections of uterine horns stimulated with estradiol benzoate from donor to recipient mice	♀C57BL/6 mice	Abdominal mechanical hyperalgesia (von Frey filaments); hind paw heat hyperalgesia (paw withdrawal, flinching/licking)^58,143^ Periorbital mechanical allodynia (von Frey filaments)^143^
Endometriosis^151^	Transgenic Lactotransferrin-Cre (LtfCre) system-specific PIK3CAH1047R activation and ARID1A deletion in endometrial epithelial cells	♀CD-1 mice	Abdominal distension, signs of severe illness (dehydration, hunching, jaundice, ruffled fur, signs of infection or nonresponsiveness)
Adenomyosis^52^	Surgical Mechanical puncture of uterine horn with a fine needle	♀BALB/c mice^52^	Not evaluated
Adenomyosis^164^	Chemical Oral administration of tamoxifen from postnatal day 2 to day 5	♀neonatal ICR mice	Hind paw heat hyperalgesia
Adenomyosis^105,108^	Biological Pituitary engraftment model induced by intrauterine pituitary transplantation	♀(SLNxC3H/He)F1mice^108^ ♀C57BL/6 mice^105^	Not evaluated
Adenomyosis^28,76,141,163^	Biological Implant of human adenomyotic lesions into immunodeficient mice	♀NOD-SCID mice,^28,76^ ♀nude mice,^141^ ♀BALB-c nu/nu mice^163^	Not evaluated^28,76,141^ Hind paw heat hyperalgesia formalin tests, spontaneous or evoked responses (licking flicking hind paws, jolting or jumping off the hot plate)^163^
Adenomyosis^110,142^	Transgenic Cre recombinase approach involving stabilized β-catenin expression in the uterus	♀PR-Cre mice crossed with *Ctnnb1*^*f(ex3)/+*^ mice^110^ ♀*Ctnnb1*^*tm1Mmt*^ mice^142^	Not evaluated^110,142^
Dysmenorrhea^29,80,153,157^	Chemical Primary dysmenorrhea induced by uterine contractions caused by oxytocin injection (i.p.) following pretreatment with estradiol benzoate (i.p.)	♀Swiss mice^80^ ♀/♂Swiss mice^157^ ♀ Sprague-Dawley rats^29,153^ ♀ICR mice^153^	Abdominal writhing by oxytocin (contractions of the abdominal wall, pelvic rotation, followed by extensor posterior limbs) (80. Yang, 2015 #14) or abdominal writhing by acetic acid and oxytocin (i.p.)^29,153^
Dysmenorrhea^14,63,97,159,162^	Chemical Secondary dysmenorrhea induced by injecting into the uterus: LPS^14^,^162^; *E. coli*,^97^ *S. aureus*^63^	♀Kunming mice,^97,162^ ♀C57BL/6 mice^14,63^ ♀BALB-c mice^159^	Not evaluated
Vulvodynia^57^	Chemical Vulvovaginal inoculations of *C.albicans*	♀CD-1 mice	Mechanical sensitivity (von Frey filaments applied to the vulva)
Vulvodynia^10^	Chemical Vulvar zymosan administration	♀Sprague-Dawley rats	Mechanical sensitivity (von Frey filaments applied to the vulva) Heat sensitivity (hot plate chamber paw/vulva lickings, jumping, earing)
Vulvodynia^22,23,133^	Chemical CFA injections at the distal level of the uterus^133^ or into the posterior perivaginal vestibular tissue (s.c.)^22,23^	♀C57BL/6 mice^22,133^ ♀Sprague-Dawley rats^23^	Not evaluated^133^ Visceromotor response to vaginal distension (abdominal muscle contraction by recording the electrical activity)^22^ Perivaginal mechanical sensitivity (Von Frey test)^23^
Vulvodynia^93^	Chemical Repeated oxazolone administration	♀ND4 Swiss mice	Tactile sensitivity in the ano-genital ridge of mice (Von Frey test)
Uterine leiomyomas^62,73^	Biological Subcutaneous implant of human fibroid fragments with estrogen supplementation	♀SCID/SCID CB17^73^ ♀ SCID mice^62^	Not evaluated
Uterine leiomyomas^51,140^	Biological Orthotopic tumors obtained by intrauterine injections of uncultured primary human fibroid cells	♀SCID/beige mice^51^ ♀BALB/c nude mice^140^	Not evaluated
Uterine leiomyomas^54^	Transgenic Spontaneous leiomyomas due to a germline mutation in the *Tsc2* gene	♀Long-Evans rats^54^	Not evaluated
Uterine leiomyomas^123^	Transgenic CaBP9K transgenic mice	♀*CaBP9K* transgenic mice^123^	Not evaluated
Pelvic inflammatory disease^111^	Chemical LPS applications after HCl intracervical administration^111^	♀C57BL/6J mice	Not evaluated
Pelvic inflammatory disease^79,149^	Biological Injections of bacterial mixture into uterine horns: *Mycoplasma urealyticum*^149^ or *Neisseria gonorrhoeae*^79^	♀Sprague-Dawley rats^149^ ♀FVB mice^79^	Not evaluated

CFA, complete Freund's adjuvant; HCl, hydrochloric acid; i.p., intraperitoneal; s.c, subcutaneous; LPS, lipopolysaccharide.

## Conflict of interest statement

The authors have no conflict of interest to declare.

## References

[1] Gonadotropin Releasing Hormone (GnRH) Analogues. LiverTox: clinical and research information on drug-induced liver injury. Bethesda (MD): National Institute of Diabetes and Digestive and Kidney Diseases, 2012. 31643176

[2] ACOG Committee Opinion No. 760. Dysmenorrhea and endometriosis in the adolescent. Obstet Gynecol 2018;132:e249–e258. 30461694 10.1097/AOG.0000000000002978

[3] Abbott J Hawe J Hunter D Holmes M Finn P Garry R. Laparoscopic excision of endometriosis: a randomized, placebo-controlled trial. Fertil Steril 2004;82:878–84. 15482763 10.1016/j.fertnstert.2004.03.046

[4] Adamson GD. Diagnosis and clinical presentation of endometriosis. Am J Obstet Gynecol 1990;162:568–9. 2137970 10.1016/0002-9378(90)90431-6

[5] Afshar Y Hastings J Roqueiro D Jeong JW Giudice LC Fazleabas AT. Changes in eutopic endometrial gene expression during the progression of experimental endometriosis in the baboon, Papio anubis. Biol Reprod 2013;88:44. 23284138 10.1095/biolreprod.112.104497 PMC3589234

[6] Akiyama Y Yao JR Kreder KJ O'Donnell MA Lutgendorf SK Lyu D Maeda D Kume H Homma Y Luo Y. Autoimmunity to urothelial antigen causes bladder inflammation, pelvic pain, and voiding dysfunction: a novel animal model for Hunner-type interstitial cystitis. Am J Physiol Renal Physiol 2021;320:F174–F182. 33308017 10.1152/ajprenal.00290.2020 PMC7948122

[7] Anjum AD Payne LN Appleby EC. Spontaneous occurrence and experimental induction of leiomyoma of the ventral ligament of the oviduct of the hen. Res Vet Sci 1988;45:341–8. 2975030

[8] Arici A Seli E Zeyneloglu HB Senturk LM Oral E Olive DL. Interleukin-8 induces proliferation of endometrial stromal cells: a potential autocrine growth factor. J Clin Endocrinol Metab 1998;83:1201–5. 9543141 10.1210/jcem.83.4.4743

[9] Attar E Tokunaga H Imir G Yilmaz MB Redwine D Putman M Gurates B Attar R Yaegashi N Hales DB Bulun SE. Prostaglandin E2 via steroidogenic factor-1 coordinately regulates transcription of steroidogenic genes necessary for estrogen synthesis in endometriosis. J Clin Endocrinol Metab 2009;94:623–31. 19001523 10.1210/jc.2008-1180 PMC2646521

[10] Awad-Igbaria Y Edelman D Ianshin E Abu-Ata S Shamir A Bornstein J Palzur E. Inflammation-induced mast cell-derived nerve growth factor: a key player in chronic vulvar pain? Brain 2025;148:331–46. 39001871 10.1093/brain/awae228

[11] Baird DD Dunson DB Hill MC Cousins D Schectman JM. High cumulative incidence of uterine leiomyoma in black and white women: ultrasound evidence. Am J Obstet Gynecol 2003;188:100–7. 12548202 10.1067/mob.2003.99

[12] Baldi A Lanza A Menicagli F Signorile PG Spugnini EP. Histological and immunohistochemical characterization of a case of endometriosis in a Guinea pig (Cavia tschudii). Case Rep Vet Med 2017;2017:4594510. 29955430 10.1155/2017/4594510 PMC6005282

[13] Ballard KD Seaman HE de Vries CS Wright JT. Can symptomatology help in the diagnosis of endometriosis? Findings from a national case-control study—part 1. BJOG 2008;115:1382–91. 18715240 10.1111/j.1471-0528.2008.01878.x

[14] Bao H Cong J Qu Q He S Zhao D Zhao H Yin S Ma D. Rosiglitazone alleviates LPS-induced endometritis via suppression of TLR4-mediated NF-kappaB activation. PLoS One 2024;19:e0280372. 38547218 10.1371/journal.pone.0280372 PMC10977739

[15] Barrier BF Allison J Hubbard GB Dick EJ Jr Brasky KM Schust DJ. Spontaneous adenomyosis in the chimpanzee (pan troglodytes): a first report and review of the primate literature: case report. Hum Reprod 2007;22:1714–7. 17452396 10.1093/humrep/dem038

[16] Barrier BF Bates GW Leland MM Leach DA Robinson RD Propst AM. Efficacy of anti-tumor necrosis factor therapy in the treatment of spontaneous endometriosis in baboons. Fertil Steril 2004;81:775–9. 15019808 10.1016/j.fertnstert.2003.09.034

[17] Baskin GB Smith SM Marx PA. Endometrial hyperplasia, polyps, and adenomyosis associated with unopposed estrogen in rhesus monkeys (Macaca mulatta). Vet Pathol 2002;39:572–5. 12243467 10.1354/vp.39-5-572

[18] Bergeron S Reed BD Wesselmann U Bohm-Starke N. Vulvodynia. Nat Rev Dis Primers 2020;6:36. 32355269 10.1038/s41572-020-0164-2

[19] Bornstein J Goldschmid N Sabo E. Hyperinnervation and mast cell activation may be used as histopathologic diagnostic criteria for vulvar vestibulitis. Gynecol Obstet Invest 2004;58:171–8. 15249746 10.1159/000079663

[20] Bornstein J Palzur E Swash M Petros P. Vulvodynia: a neuroinflammatory pain syndrome originating in pelvic visceral nerve plexuses due to mechanical factors. Arch Gynecol Obstet 2022;306:1411–5. 35147761 10.1007/s00404-022-06424-4 PMC9519726

[21] Cardaillac C Levesque A Riant T Mortier A Neunlist M Perrouin-Verbe MA Volteau C Thubert T Brochard C Ploteau S. Evaluation of a scoring system for the detection of central sensitization among women with chronic pelvic pain. Am J Obstet Gynecol 2023;229:530.e1–530.e17. 10.1016/j.ajog.2023.07.044 37516398

[22] Castro J Harrington AM Chegini F Matusica D Spencer NJ Brierley SM Haberberger RV Barry CM. Clodronate treatment prevents vaginal hypersensitivity in a mouse model of Vestibulodynia. Front Cell Infect Microbiol 2022;11:784972. 35118009 10.3389/fcimb.2021.784972 PMC8803747

[23] Chakrabarty A Liao Z Mu Y Smith PG. Inflammatory renin-angiotensin system disruption attenuates sensory hyperinnervation and mechanical hypersensitivity in a rat model of provoked vestibulodynia. The J Pain 2018;19:264–77. 29155208 10.1016/j.jpain.2017.10.006 PMC5811351

[24] Chan WY Hill JC. Determination of menstrual prostaglandin levels in non-dysmenorrheic and dysmenorrheic subjects. Prostaglandins 1978;15:365–75. 635225 10.1016/0090-6980(78)90176-4

[25] Chapron C Vannuccini S Santulli P Abrão MS Carmona F Fraser IS Gordts S Guo SW Just PA Noël JC Pistofidis G Van den Bosch T Petraglia F. Diagnosing adenomyosis: an integrated clinical and imaging approach. Hum Reprod Update 2020;26:392–411. 32097456 10.1093/humupd/dmz049

[26] Chavda V Joshi B Bhatt AD Patel S Savaliya M Chaurasia B. Persistent pelvic pain in women: unraveling diagnostic challenges and the overlooked role of pelvic congestion syndrome. Ann Med Surg 2025;87:7950–2. 10.1097/MS9.0000000000004272 PMC12688801 41377464

[27] Chen Q Li YW Wang S Fan QB Shi HH Leng JH Sun DW Lang JH Zhu L. Clinical manifestations of adenomyosis patients with or without pain symptoms. J Pain Res 2019;12:3127–33. 31814754 10.2147/JPR.S212117 PMC6861517

[28] Chen YJ Li HY Huang CH Twu NF Yen MS Wang PH Chou TY Liu YN Chao KC Yang MH. Oestrogen-induced epithelial-mesenchymal transition of endometrial epithelial cells contributes to the development of adenomyosis. J Pathol 2010;222:261–70. 20814901 10.1002/path.2761

[29] Chiang YF Hung HC Chen HY Huang KC Lin PH Chang JY Huang TC Hsia SM. The inhibitory effect of extra virgin olive oil and its active compound oleocanthal on prostaglandin-induced uterine hypercontraction and pain-ex vivo and in vivo study. Nutrients 2020;12.3012. 33008039 10.3390/nu12103012 PMC7599558

[30] Cianciolo RE Butler SD Eggers JS Dick EJ Jr Leland MM de la Garza M Brasky KM Cummins LB Hubbard GB. Spontaneous neoplasia in the baboon (papio spp.). J Med Primatol 2007;36:61–79. 17493137 10.1111/j.1600-0684.2006.00202.x

[31] Clemenza S Vannuccini S Capezzuoli T Meleca CI Pampaloni F Petraglia F. Is primary dysmenorrhea a precursor of future endometriosis development? Gynecol Endocrinol 2021;37:287–93. 33569996 10.1080/09513590.2021.1878134

[32] Cohen J Naoura I Castela M Von N'Guyen T Oster M Fontaine R Chabbert-Buffet N Darai E Aractingi S. Pregnancy affects morphology of induced endometriotic lesions in a mouse model through alteration of proliferation and angiogenesis. Eur J Obstet Gynecol Reprod Biol 2014;183:70–7. 25461356 10.1016/j.ejogrb.2014.10.038

[33] Cook AL Rogers TD Sowers M. Spontaneous uterine leiomyosarcoma in a rhesus macaque. Contemp Top Lab Anim Sci 2004;43:47–9. 14984291

[34] Craft RM Mogil JS Aloisi AM. Sex differences in pain and analgesia: the role of gonadal hormones. Eur J Pain 2004;8:397–411. 15324772 10.1016/j.ejpain.2004.01.003

[35] Cummings AM Metcalf JL. Induction of endometriosis in mice: a new model sensitive to estrogen. Reprod Toxicol 1995;9:233–8. 7579907 10.1016/0890-6238(95)00004-t

[36] D'Hooghe TM Bambra CS Cornillie FJ Isahakia M Koninckx PR. Prevalence and laparoscopic appearance of spontaneous endometriosis in the baboon (Papio anubis, Papio cynocephalus). Biol Reprod 1991;45:411–6. 1838282 10.1095/biolreprod45.3.411

[37] D'Hooghe TM Kyama CM Chai D Fassbender A Vodolazkaia A Bokor A Mwenda JM. Nonhuman primate models for translational research in endometriosis. Reprod Sci 2009;16:152–61. 19208783 10.1177/1933719108322430

[38] D'Hooghe TM Nugent NP Cuneo S Chai DC Deer F Debrock S Kyama CM Mihalyi A Mwenda JM. Recombinant human TNFRSF1A (r-hTBP1) inhibits the development of endometriosis in baboons: a prospective, randomized, placebo- and drug-controlled study. Biol Reprod 2006;74:131–6. 16177224 10.1095/biolreprod.105.043349

[39] De Logu F Boccella S Guida F. Editorial: the role of neuroinflammation in chronic pain development and maintenance. Front Pharmacol 2021;12:821534. 35002746 10.3389/fphar.2021.821534 PMC8733678

[40] De Logu F Li Puma S Landini L Tuccinardi T Poli G Preti D De Siena G Patacchini R Tsagareli MG Geppetti P Nassini R. The acyl-glucuronide metabolite of ibuprofen has analgesic and anti-inflammatory effects via the TRPA1 channel. Pharmacol Res 2019;142:127–39. 30794923 10.1016/j.phrs.2019.02.019

[41] De Logu F Marini M Landini L Souza Monteiro de Araujo D Bartalucci N Trevisan G Bruno G Marangoni M Schmidt BL Bunnett NW Geppetti P Nassini R. Peripheral nerve resident macrophages and schwann cells mediate cancer-induced pain. Cancer Res 2021;81:3387–401. 33771895 10.1158/0008-5472.CAN-20-3326 PMC8260461

[42] De Logu F Nassini R Hegron A Landini L Jensen DD Latorre R Ding J Marini M Souza Monteiro de Araujo D Ramirez-Garcia P Whittaker M Retamal J Titiz M Innocenti A Davis TP Veldhuis N Schmidt BL Bunnett NW Geppetti P. Schwann cell endosome CGRP signals elicit periorbital mechanical allodynia in mice. Nat Commun 2022;13:646. 35115501 10.1038/s41467-022-28204-z PMC8813987

[43] De Logu F Nassini R Materazzi S Carvalho Goncalves M Nosi D Rossi Degl'Innocenti D Marone IM Ferreira J Li Puma S Benemei S Trevisan G Souza Monteiro de Araujo D Patacchini R Bunnett NW Geppetti P. Schwann cell TRPA1 mediates neuroinflammation that sustains macrophage-dependent neuropathic pain in mice. Nat Commun 2017;8:1887. 29192190 10.1038/s41467-017-01739-2 PMC5709495

[44] Di Girolamo G Sanchez AJ De Los Santos AR Gonzalez CD. Is acetaminophen, and its combination with pamabrom, an effective therapeutic option in primary dysmenorrhoea? Expert Opin Pharmacother 2004;5:561–70. 15013925 10.1517/14656566.5.3.561

[45] Diaz I Lumsden MA Zeppernick M. Medical treatment of fibroids: FIGO best practice guidance. Int J Gynecol Obstet 2025;171:507–12. 10.1002/ijgo.70497 PMC12553110 40927887

[46] Dick EJ Jr Hubbard GB Martin LJ Leland MM. Record review of baboons with histologically confirmed endometriosis in a large established colony. J Med Primatol 2003;32:39–47. 12733601 10.1034/j.1600-0684.2003.00008.x

[47] DiGiacomo RF. Gynecologic pathology in the rhesus monkey (macaca mulatta). II. Findings in laboratory and free-ranging monkeys. Vet Pathol 1977;14:539–46. 412291 10.1177/030098587701400601

[48] Dolmans MM Petraglia F Catherino WH Donnez J. Pathogenesis of uterine fibroids: current understanding and future directions. Fertil Steril 2024;122:6–11. 38453042 10.1016/j.fertnstert.2024.02.048

[49] Donnez J Stratopoulou CA Dolmans MM. Endometriosis and adenomyosis: similarities and differences. Best Pract Res Clin Obstet Gynaecol 2024;92:102432. 38103509 10.1016/j.bpobgyn.2023.102432

[50] Dorning A Dhami P Panir K Hogg C Park E Ferguson GD Hargrove D Karras J Horne AW Greaves E. Bioluminescent imaging in induced mouse models of endometriosis reveals differences in four model variations. Dis Models Mech 2021;14:dmm049070. 10.1242/dmm.049070 PMC8419713 34382636

[51] Drosch M Bullerdiek J Zollner TM Prinz F Koch M Schmidt N. A novel mouse model that closely mimics human uterine leiomyomas. Fertil Steril 2013;99:927–35.e6. 23260859 10.1016/j.fertnstert.2012.11.032

[52] Elsherbini M Koga K Hiraoka T Kumasawa K Maki E Satake E Taguchi A Makabe T Takeuchi A Izumi G Takamura M Harada M Hirata T Hirota Y Wada-Hiraike O Osuga Y. Establishment of a novel mouse model of adenomyosis suitable for longitudinal and quantitative analysis and perinatal outcome studies. Sci Rep 2022;12:17515. 36266437 10.1038/s41598-022-22413-8 PMC9585053

[53] Eschenbach DA Wolner-Hanssen P Hawes SE Pavletic A Paavonen J Holmes KK. Acute pelvic inflammatory disease: associations of clinical and laboratory findings with laparoscopic findings. Obstet Gynecol 1997;89:184–92. 9015018 10.1016/S0029-7844(96)00429-2

[54] Everitt JI Wolf DC Howe SR Goldsworthy TL Walker C. Rodent model of reproductive tract leiomyomata. Clinical and pathological features. Am J Pathol 1995;146:1556–67. 7778693 PMC1870902

[55] Falsetta ML Maddipati KR Honn KV. Inflammation, lipids, and pain in vulvar disease. Pharmacol Ther 2023;248:108467. 37285943 10.1016/j.pharmthera.2023.108467 PMC10527276

[56] Fan L Liu Z Zhang Z Li T Li H Chen J Zong X Zhang X Chen X Bai H Wang F Shang C. Identifying the clinical presentations, progression, and sequela of pelvic inflammatory disease through physiological, histological and ultrastructural evaluation of a rat animal model. Ann Transl Med 2021;9:1710. 35071404 10.21037/atm-21-3345 PMC8743706

[57] Farmer MA Taylor AM Bailey AL Tuttle AH MacIntyre LC Milagrosa ZE Crissman HP Bennett GJ Ribeiro-da-Silva A Binik YM Mogil JS. Repeated vulvovaginal fungal infections cause persistent pain in a mouse model of vulvodynia. Sci Transl Med 2011;3:101ra191. 10.1126/scitranslmed.3002613 PMC3243907 21937756

[58] Fattori V Franklin NS Gonzalez-Cano R Peterse D Ghalali A Madrian E Verri WA Jr Andrews N Woolf CJ Rogers MS. Nonsurgical mouse model of endometriosis-associated pain that responds to clinically active drugs. PAIN 2020;161:1321–31. 32132396 10.1097/j.pain.0000000000001832

[59] Ferries-Rowe E Corey E Archer JS. Primary dysmenorrhea: diagnosis and therapy. Obstet Gynecol 2020;136:1047–58. 33030880 10.1097/AOG.0000000000004096

[60] Fiçicioğlu C Tekin HI Arioğlu PF Okar I. A murine model of adenomyosis: the effects of hyperprolactinemia induced by fluoxetine hydrochloride, a selective serotonin reuptake inhibitor, on adenomyosis induction in wistar albino rats. Acta Eur Fertil 1995;26:75–9. 9098464

[61] Field KJ Griffith JW Lang CM. Spontaneous reproductive tract leiomyomas in aged guinea-pigs. J Comp Pathol 1989;101:287–94. 2584448 10.1016/0021-9975(89)90038-8

[62] Fritsch M Schmidt N Groticke I Frisk AL Keator CS Koch M Slayden OD. Application of a patient derived xenograft model for predicative study of uterine fibroid disease. PLoS One 2015;10:e0142429. 26588841 10.1371/journal.pone.0142429 PMC4654507

[63] Gao S Gao Y Cai L Qin R. Luteolin attenuates staphylococcus aureus-induced endometritis through inhibiting ferroptosis and inflammation via activating the Nrf2/GPX4 signaling pathway. Microbiol Spectr 2024;12:e0327923. 38169293 10.1128/spectrum.03279-23 PMC10846197

[64] Gelberg HB McEntee K. Pathology of the canine and feline uterine tube. Vet Pathol 1986;23:770–5. 3811142 10.1177/030098588602300617

[65] Gemesi TS Lum D Atashroo D Chao L. Minimally invasive and ablative therapies for symptomatic uterine fibroids: a narrative review. Curr Opin Obstet Gynecol 2025;37:346–52. 40742981 10.1097/GCO.0000000000001056

[66] Ghant MS Sengoba KS Recht H Cameron KA Lawson AK Marsh EE. Beyond the physical: a qualitative assessment of the burden of symptomatic uterine fibroids on women's emotional and psychosocial health. J Psychosom Res 2015;78:499–503. 25725565 10.1016/j.jpsychores.2014.12.016

[67] Giudice LC. Clinical practice. Endometriosis. N Engl J Med 2010;362:2389–98. 20573927 10.1056/NEJMcp1000274 PMC3108065

[68] Giuliani E As-Sanie S Marsh EE. Epidemiology and management of uterine fibroids. Int J Gynecol Obstet 2020;149:3–9. 10.1002/ijgo.13102 31960950

[69] Goetsch MF Morgan TK Korcheva VB Li H Peters D Leclair CM. Histologic and receptor analysis of primary and secondary vestibulodynia and controls: a prospective study. Am J Obstet Gynecol 2010;202:614.e1–614.e8. 10.1016/j.ajog.2010.01.028 20430353

[70] Greaves E Cousins FL Murray A Esnal-Zufiaurre A Fassbender A Horne AW Saunders PT. A novel mouse model of endometriosis mimics human phenotype and reveals insights into the inflammatory contribution of shed endometrium. Am J Pathol 2014;184:1930–9. 24910298 10.1016/j.ajpath.2014.03.011 PMC4076466

[71] Greaves P White IN. Experimental adenomyosis. Best Pract Res Clin Obstet Gynaecol 2006;20:503–10. 16500151 10.1016/j.bpobgyn.2006.01.003

[72] Halbedl H Pfabigan DM Ebhardt I Trutnovsky G. Mindfulness-based body scan training in multimodal physiotherapy for vulvodynia—a randomized controlled feasibility study. J Psychosom Obstet Gynecol 2025;46:2531057. 10.1080/0167482X.2025.2531057 40653877

[73] Hassan MH Eyzaguirre E Arafa HM Hamada FM Salama SA Al-Hendy A. Memy I: a novel murine model for uterine leiomyoma using adenovirus-enhanced human fibroid explants in severe combined immune deficiency mice. Am J Obstet Gynecol 2008;199:156.e1–156.e8. 10.1016/j.ajog.2008.02.010 PMC4272582 18468574

[74] Hayashi S Nakamura T Motooka Y Ito F Jiang L Akatsuka S Iwase A Kajiyama H Kikkawa F Toyokuni S. Novel ovarian endometriosis model causes infertility via iron-mediated oxidative stress in mice. Redox Biol 2020;37:101726. 32961443 10.1016/j.redox.2020.101726 PMC7509075

[75] Hong DG Hwang SM Park JM. Efficacy of ganglion impar block on vulvodynia: case series and results of mid- and long-term follow-up. Medicine (Baltimore) 2021;100:e26799. 34397737 10.1097/MD.0000000000026799 PMC8322564

[76] Huang TS Chen YJ Chou TY Chen CY Li HY Huang BS Tsai HW Lan HY Chang CH Twu NF Yen MS Wang PH Chao KC Lee CC Yang MH. Oestrogen-induced angiogenesis promotes adenomyosis by activating the Slug-VEGF axis in endometrial epithelial cells. J Cell Mol Med 2014;18:1358–71. 24758741 10.1111/jcmm.12300 PMC4124020

[77] Iacovides S Avidon I Baker FC. What we know about primary dysmenorrhea today: a critical review. Hum Reprod Update 2015;21:762–78. 26346058 10.1093/humupd/dmv039

[78] Ishikawa H Ishi K Serna VA Kakazu R Bulun SE Kurita T. Progesterone is essential for maintenance and growth of uterine leiomyoma. Endocrinology 2010;151:2433–42. 20375184 10.1210/en.2009-1225 PMC2875812

[79] Islam EA Shaik-Dasthagirisaheb Y Kaushic C Wetzler LM Gray-Owen SD. The reproductive cycle is a pathogenic determinant during gonococcal pelvic inflammatory disease in mice. Mucosal Immunol 2016;9:1051–64. 26693700 10.1038/mi.2015.122 PMC4915993

[80] Jesuino F Reis JP Whitaker JCP Campos A Pastor MVD Cechinel Filho V Quintao NLM. Effect of synadenium grantii and its isolated compound on dysmenorrhea behavior model in mice. Inflammopharmacology 2019;27:613–20. 29948493 10.1007/s10787-018-0501-1

[81] Ji RR Nackley A Huh Y Terrando N Maixner W. Neuroinflammation and central sensitization in chronic and widespread pain. Anesthesiology 2018;129:343–66. 29462012 10.1097/ALN.0000000000002130 PMC6051899

[82] Jossens MO Sweet RL. Pelvic inflammatory disease: risk factors and microbial etiologies. J Obstet Gynecol Neonatal Nurs 1993;22:169–79. 10.1111/j.1552-6909.1993.tb01796.x 8478740

[83] Ju H Jones M Mishra G. The prevalence and risk factors of dysmenorrhea. Epidemiologic Rev 2014;36:104–13. 10.1093/epirev/mxt009 24284871

[84] Kanellopoulos D Karagianni D Pergialiotis V Nikiteas N Lazaris AC Iliopoulos D. The effect of endometriosis on fertility in an animal model. J Med Life 2022;15:1170–5. 36415526 10.25122/jml-2021-0391 PMC9635238

[85] Kaspareit J Friderichs-Gromoll S Buse E Habermann G. Spontaneous neoplasms observed in cynomolgus monkeys (Macaca fascicularis) during a 15-year period. Exp Toxicol Pathol 2007;59:163–9. 17869495 10.1016/j.etp.2007.06.001

[86] Keogh E Attridge N Walsh J Bartlett J Francis R Bultitude JH Eccleston C. Attentional biases towards body expressions of pain in men and women. J Pain 2021;22:1696–708. 34174386 10.1016/j.jpain.2021.06.003

[87] Keppel Hesselink JM Kopsky DJ Sajben N. New topical treatment of vulvodynia based on the pathogenetic role of cross talk between nociceptors, immunocompetent cells, and epithelial cells. J Pain Res 2016;9:757–62. 27757050 10.2147/JPR.S115407 PMC5055105

[88] Kim DJ Ki YJ Jang BH Kim S Kim SH Jung KT. Clinically relevant concentrations of dexmedetomidine May reduce oxytocin-induced myometrium contractions in pregnant rats. Anesth Pain Med (Seoul) 2020;15:451–8. 33329848 10.17085/apm.20036 PMC7724122

[89] Kirsch E Rahman S Kerolus K Hasan R Kowalska DB Desai A Bergese SD. Dysmenorrhea, a narrative review of therapeutic options. J Pain Res 2024;17:2657–66. 39161419 10.2147/JPR.S459584 PMC11332412

[90] Kiviat NB Wolner-Hanssen P Eschenbach DA Wasserheit JN Paavonen JA Bell TA Critchlow CW Stamm WE Moore DE Holmes KK. Endometrial histopathology in patients with culture-proved upper genital tract infection and laparoscopically diagnosed acute salpingitis. Am J Surg Pathol 1990;14:167–75. 2137304 10.1097/00000478-199002000-00008

[91] Lamvu G Carrillo J Ouyang C Rapkin A. Chronic pelvic pain in women: a review. JAMA 2021;325:2381–91. 34128995 10.1001/jama.2021.2631

[92] Landini L Marini M Souza Monteiro de Araujo D Romitelli A Montini M Albanese V Titiz M Innocenti A Bianchini F Geppetti P Nassini R De Logu F. Schwann cell insulin-like growth factor receptor type-1 mediates metastatic bone cancer pain in mice. Brain Behav Immun 2023;110:348–64. 36940752 10.1016/j.bbi.2023.03.013

[93] Landry J Martinov T Mengistu H Dhanwada J Benck CJ Kline J Boo B Swanson L Tonc E Daughters R Fife BT Chatterjea D. Repeated hapten exposure induces persistent tactile sensitivity in mice modeling localized provoked vulvodynia. PLoS One 2017;12:e0169672. 28158195 10.1371/journal.pone.0169672 PMC5291437

[94] Latremoliere A Woolf CJ. Central sensitization: a generator of pain hypersensitivity by central neural plasticity. J Pain 2009;10:895–926. 19712899 10.1016/j.jpain.2009.06.012 PMC2750819

[95] Latthe P Latthe M Say L Gulmezoglu M Khan KS. WHO systematic review of prevalence of chronic pelvic pain: a neglected reproductive health morbidity. BMC Public Health 2006;6:177. 16824213 10.1186/1471-2458-6-177 PMC1550236

[96] Lee SE Greenough EK Oancea P Scheinfeld AR Douglas AM Gaudet AD. Sex differences in pain: spinal cord injury in female and Male mice elicits behaviors related to neuropathic pain. J Neurotrauma 2023;40:833–44. 36719772 10.1089/neu.2022.0482

[97] Li Z Shi L Li Q Zhao J Lu W Wang J. The expression and bioinformatics analysis of circular RNAs in endometritis mouse uterus tissues. Molecules 2022;27:3682. 35744807 10.3390/molecules27123682 PMC9230989

[98] Long CT Luong RH McKeon GP Albertelli M. Uterine leiomyoma in a Guyanese squirrel monkey (Saimiri sciureus sciureus). J Am Assoc Lab Anim Sci 2010;49:226–30. 20353700 PMC2846013

[99] Machado SA Bahr JM Hales DB Braundmeier AG Quade BJ Nowak RA. Validation of the aging hen (gallus Gallus domesticus) as an animal model for uterine leiomyomas. Biol Reprod 2012;87:86. 22811571 10.1095/biolreprod.112.101188 PMC4434995

[100] MacKenzie WF Casey HW. Animal model of human disease. Endometriosis. Animal model: endometriosis in rhesus monkeys. Am J Pathol 1975;80:341–4. 1163633 PMC1912929

[101] Marquardt RM Jeong JW Fazleabas AT. Animal models of adenomyosis. Semin Reprod Med 2020;38:168–78. 33105508 10.1055/s-0040-1718741 PMC8054228

[102] Maruo T Laoag-Fernandez JB Pakarinen P Murakoshi H Spitz IM Johansson E. Effects of the levonorgestrel-releasing intrauterine system on proliferation and apoptosis in the endometrium. Hum Reprod 2001;16:2103–8. 11574499 10.1093/humrep/16.10.2103

[103] McEntee K Nielsen SW. Tumours of the female genital tract. Bull World Health Organ 1976;53:217–26. 1086152 PMC2366500

[104] Mendiratta V Lentz GM. 35—primary and secondary dysmenorrhea, premenstrual syndrome, and premenstrual dysphoric disorder: etiology, diagnosis, management. In: Gershenson DM Lentz GM Valea FA Lobo RA, editors. Comprehensive gynecology. 8th ed. St. Louis: Elsevier, 2022. pp. 768–80.e764.

[105] Mori T Nagasawa H Takahashi S. The induction of adenomyosis in mice by intrauterine pituitary isografts. Life Sci 1981;29:1277–82. 7300555 10.1016/0024-3205(81)90234-4

[106] Morotti M Vincent K Becker CM. Mechanisms of pain in endometriosis. Eur J Obstet Gynecol Reprod Biol 2017;209:8–13. 27522645 10.1016/j.ejogrb.2016.07.497

[107] Mozzachio K Linder K Dixon D. Uterine smooth muscle tumors in potbellied pigs (sus scrofa) resemble human fibroids: a potential animal model. Toxicol Pathol 2004;32:402–7. 15307213 10.1080/01926230490440880

[108] Nagasawa H Naito T. Enhanced potentials for mammary tumourigenesis and uterine adenomyosis in (SLN x C3H/He)F1 virgin mice. Lab Anim 1992;26:23–4. 1548842 10.1258/002367792780809084

[109] Nie J Liu X Guo SW. Immunoreactivity of oxytocin receptor and transient receptor potential vanilloid type 1 and its correlation with dysmenorrhea in adenomyosis. Am J Obstet Gynecol 2010;202:346.e1–346.e8. 10.1016/j.ajog.2009.11.035 20096818

[110] Oh SJ Shin JH Kim TH Lee HS Yoo JY Ahn JY Broaddus RR Taketo MM Lydon JP Leach RE Lessey BA Fazleabas AT Lim JM Jeong JW. β-Catenin activation contributes to the pathogenesis of adenomyosis through epithelial-mesenchymal transition. J Pathol 2013;231:210–22. 23784889 10.1002/path.4224 PMC4105844

[111] Oh Y Lee J Kim HC Hahn TW Yoon BI Han JH Kwon YS Park JJ Koo DB Rhee KJ Jung BD. Establishment of hydrochloric acid/lipopolysaccharide-induced pelvic inflammatory disease model. J Vet Sci 2016;17:413–9. 26726020 10.4142/jvs.2016.17.3.413 PMC5037311

[112] Park KS Park KI Hwang DS Lee JM Jang JB Lee CH. A review of in vitro and in vivo studies on the efficacy of herbal medicines for primary dysmenorrhea. Evid Based Complement Altern Med 2014;2014:296860. 10.1155/2014/296860 PMC4238180 25431607

[113] Patton DL Cosgrove Sweeney YT Paul KJ. A summary of preclinical topical microbicide vaginal safety and chlamydial efficacy evaluations in a pigtailed macaque model. Sex Transm Dis 2008;35:889–97. 18607310 10.1097/OLQ.0b013e31817dfdb8

[114] Patton DL Kuo CC Wang SP Halbert SA. Distal tubal obstruction induced by repeated Chlamydia trachomatis salpingeal infections in pig-tailed macaques. J Infect Dis 1987;155:1292–9. 3572039 10.1093/infdis/155.6.1292

[115] Patton DL Sweeney YT Stamm WE. Significant reduction in inflammatory response in the macaque model of chlamydial pelvic inflammatory disease with azithromycin treatment. J Infect Dis 2005;192:129–35. 15942902 10.1086/431365

[116] Patton DL Wølner-Hanssen P Cosgrove SJ Holmes KK. The effects of Chlamydia trachomatis on the female reproductive tract of the Macaca nemestrina after a single tubal challenge following repeated cervical inoculations. Obstet Gynecol 1990;76:643–50. 2216196

[117] Peitsidis P Koukoulomati A. Tranexamic acid for the management of uterine fibroid tumors: a systematic review of the current evidence. World J Clin Cases 2014;2:893–8. 25516866 10.12998/wjcc.v2.i12.893 PMC4266839

[118] Peng Y Zheng X Fan Z Zhou H Zhu X Wang G Liu Z. Paeonol alleviates primary dysmenorrhea in mice via activating CB2R in the uterus. Phytomedicine 2020;68:153151. 32058234 10.1016/j.phymed.2019.153151

[119] Peregrino PFM Messina ML Menezes MR Júnior JMS Cerri GG Baracat EC. Magnetic resonance-guided focused ultrasound for uterine fibroids. Clinics (Sao Paulo) 2025;80:100603. 40043426 10.1016/j.clinsp.2025.100603 PMC11926681

[120] Porpora MG Koninckx PR Piazze J Natili M Colagrande S Cosmi EV. Correlation between endometriosis and pelvic pain. J Am Assoc Gynecol Laparosc 1999;6:429–34. 10548700 10.1016/s1074-3804(99)80006-1

[121] Proctor M Farquhar C. Diagnosis and management of dysmenorrhoea. BMJ 2006;332:1134–8. 16690671 10.1136/bmj.332.7550.1134 PMC1459624

[122] Reed BD Caron AM Gorenflo DW Haefner HK. Treatment of vulvodynia with tricyclic antidepressants: efficacy and associated factors. J Lower Genital Tract Dis 2006;10:245–51. 10.1097/01.lgt.0000225899.75207.0a 17012991

[123] Romagnolo B Molina T Leroy G Blin C Porteux A Thomasset M Vandewalle A Kahn A Perret C. Estradiol-dependent uterine leiomyomas in transgenic mice. J Clin Invest 1996;98:777–84. 8698870 10.1172/JCI118850 PMC507488

[124] Ross J Guaschino S Cusini M Jensen J. 2017 European guideline for the management of pelvic inflammatory disease. Int J STD AIDS 2018;29:108–14. 29198181 10.1177/0956462417744099

[125] Ryan A Healey M Cheng C Dior U Reddington C. Central sensitisation in pelvic pain: a cohort study. Aust N Z J Obstet Gynaecol 2022;62:868–74. 35950448 10.1111/ajo.13596

[126] Sadownik LA. Etiology, diagnosis, and clinical management of vulvodynia. Int J Womens Health 2014;6:437–49. 24833921 10.2147/IJWH.S37660 PMC4014358

[127] Sammour A Pirwany I Usubutun A Arseneau J Tulandi T. Correlations between extent and spread of adenomyosis and clinical symptoms. Gynecol Obstet Invest 2002;54:213–6. 12592064 10.1159/000068385

[128] Santulli P Vannuccini S Bourdon M Chapron C Petraglia F. Adenomyosis: the missed disease. Reprod Biomed Online 2025;50:104837. 40287215 10.1016/j.rbmo.2025.104837

[129] Savaris RF Fuhrich DG Maissiat J Duarte RV Ross J. Antibiotic therapy for pelvic inflammatory disease. Cochrane Database Syst Rev 2020;8:CD010285. 32820536 10.1002/14651858.CD010285.pub3 PMC8094882

[130] Schmauss C Yaksh TL. In vivo studies on spinal opiate receptor systems mediating antinociception. II. Pharmacological profiles suggesting a differential association of mu, delta and kappa receptors with visceral chemical and cutaneous thermal stimuli in the rat. J Pharmacol Exp Ther 1984;228:1–12. 6319664

[131] Schroll JB Black AY Farquhar C Chen I. Combined oral contraceptive pill for primary dysmenorrhoea. Cochrane Database Syst Rev 2023;7:CD002120. 37523477 10.1002/14651858.CD002120.pub4 PMC10388393

[132] Shakiba K Bena JF McGill KM Minger J Falcone T. Surgical treatment of endometriosis: a 7-year follow-up on the requirement for further surgery. Obstet Gynecol 2008;111:1285–92. 18515510 10.1097/AOG.0b013e3181758ec6

[133] Sharma H Ji E Yap P Vilimas P Kyloh M Spencer NJ Haberberger RV Barry CM. Innervation changes induced by inflammation in the Murine vagina. Neuroscience 2018;372:16–26. 29294338 10.1016/j.neuroscience.2017.12.026

[134] Simitsidellis I Gibson DA Saunders PTK. Animal models of endometriosis: replicating the aetiology and symptoms of the human disorder. Best Pract Res Clin Endocrinol Metab 2018;32:257–69. 29779580 10.1016/j.beem.2018.03.004

[135] Somigliana E Viganò P Rossi G Carinelli S Vignali M Panina-Bordignon P. Endometrial ability to implant in ectopic sites can be prevented by interleukin-12 in a murine model of endometriosis. Hum Reprod 1999;14:2944–50. 10601076 10.1093/humrep/14.12.2944

[136] Spoelstra SK Borg C Weijmar Schultz WC. Anticonvulsant pharmacotherapy for generalized and localized vulvodynia: a critical review of the literature. J Psychosom Obstet Gynecol 2013;34:133–8. 10.3109/0167482X.2013.823942 23952171

[137] Stewart EA Laughlin-Tommaso SK Catherino WH Lalitkumar S Gupta D Vollenhoven B. Uterine fibroids. Nat Rev Dis Primers 2016;2:16043. 27335259 10.1038/nrdp.2016.43

[138] Stöcklin-Gautschi NM Guscetti F Reichler IM Geissbühler U Braun SA Arnold S. Identification of focal adenomyosis as a uterine lesion in two dogs. J Small Anim Pract 2001;42:413–6. 11518423 10.1111/j.1748-5827.2001.tb02492.x

[139] Stringer EM De Voe RS Valea F Toma S Mulvaney G Pruitt A Troan B Loomis MR. Medical and surgical management of reproductive neoplasia in two western lowland gorillas (gorilla gorilla gorilla). J Med Primatol 2010;39:328–35. 20412375 10.1111/j.1600-0684.2010.00414.x

[140] Suzuki Y Ii M Saito T Terai Y Tabata Y Ohmichi M Asahi M. Establishment of a novel mouse xenograft model of human uterine leiomyoma. Sci Rep 2018;8:8872. 29891843 10.1038/s41598-018-27138-1 PMC5995841

[141] Suzuki-Kakisaka H Murakami T Hirano T Terada Y Yaegashi N Okamura K. Selective accumulation of PpIX and photodynamic effect after aminolevulinic acid treatment of human adenomyosis xenografts in nude mice. Fertil Steril 2008;90:1523–7. 18462731 10.1016/j.fertnstert.2007.09.001

[142] Tanwar PS Lee HJ Zhang L Zukerberg LR Taketo MM Rueda BR Teixeira JM. Constitutive activation of beta-catenin in uterine stroma and smooth muscle leads to the development of mesenchymal tumors in mice. Biol Reprod 2009;81:545–52. 19403928 10.1095/biolreprod.108.075648 PMC2731978

[143] Titiz M Landini L Souza Monteiro de Araujo D Marini M Seravalli V Chieca M Pensieri P Montini M De Siena G Pasquini B Vannuccini S Iannone LF Cunha TM Brancolini G Bellantoni E Scuffi I Mastricci A Tesi M Di Tommaso M Petraglia F Geppetti P Nassini R De Logu F. Schwann cell C5aR1 co-opts inflammasome NLRP1 to sustain pain in a mouse model of endometriosis. Nat Commun 2024;15:10142. 39587068 10.1038/s41467-024-54486-6 PMC11589863

[144] Tuo L Tang S Li S Gu S Xie Z. Murine models and research progress on dysmenorrhea. Reprod Sci 2023;30:2362–72. 37010703 10.1007/s43032-023-01220-0

[145] Tympanidis P Terenghi G Dowd P. Increased innervation of the vulval vestibule in patients with vulvodynia. Br J Dermatol 2003;148:1021–7. 12786836 10.1046/j.1365-2133.2003.05308.x

[146] Van Voorhis WC Barrett LK Sweeney YT Kuo CC Patton DL. Repeated Chlamydia trachomatis infection of Macaca nemestrina fallopian tubes produces a Th1-like cytokine response associated with fibrosis and scarring. Infect Immun 1997;65:2175–82. 9169748 10.1128/iai.65.6.2175-2182.1997 PMC175300

[147] Vannuccini S Petraglia F Carmona F Calaf J Chapron C. The modern management of uterine fibroids-related abnormal uterine bleeding. Fertil Steril 2024;122:20–30. 38723935 10.1016/j.fertnstert.2024.04.041

[148] Wang J Doan LV. Clinical pain management: current practice and recent innovations in research. Cell Rep Med 2024;5:101786. 39383871 10.1016/j.xcrm.2024.101786 PMC11513809

[149] Wei A Feng H Jia XM Tang H Liao YY Li BR. Ozone therapy ameliorates inflammation and endometrial injury in rats with pelvic inflammatory disease. Biomed Pharmacother 2018;107:1418–25. 30257358 10.1016/j.biopha.2018.07.137

[150] Willets JM Brighton PJ Mistry R Morris GE Konje JC Challiss RA. Regulation of oxytocin receptor responsiveness by G protein-coupled receptor kinase 6 in human myometrial smooth muscle. Mol Endocrinol 2009;23:1272–80. 19423652 10.1210/me.2009-0047 PMC5419184

[151] Wilson MR Holladay J Chandler RL. A mouse model of endometriosis mimicking the natural spread of invasive endometrium. Hum Reprod 2020;35:58–69. 31886851 10.1093/humrep/dez253 PMC8205619

[152] Wolner-Hanssen P Patton DL Holmes KK. Protective immunity in pig-tailed macaques after cervical infection with Chlamydia trachomatis. Sex Transm Dis 1991;18:21–5. 1827541 10.1097/00007435-199101000-00005

[153] Wong J Chiang YF Shih YH Chiu CH Chen HY Shieh TM Wang KL Huang TC Hong YH Hsia SM. Salvia sclarea L. Essential oil extract and its antioxidative phytochemical sclareol inhibit oxytocin-induced uterine hypercontraction dysmenorrhea model by inhibiting the Ca(2+)-MLCK-MLC20 signaling Cascade: an Ex vivo and in vivo study. Antioxidants (Basel) 2020;9:10. 10.3390/antiox9100991 PMC7602146 33066489

[154] Woolf CJ. Central sensitization: implications for the diagnosis and treatment of pain. PAIN 2011;152:S2-s15. 20961685 10.1016/j.pain.2010.09.030 PMC3268359

[155] Xie AX Iguchi N Clarkson TC Malykhina AP. Pharmacogenetic inhibition of lumbosacral sensory neurons alleviates visceral hypersensitivity in a mouse model of chronic pelvic pain. PLoS One 2022;17:e0262769. 35077502 10.1371/journal.pone.0262769 PMC8789164

[156] Xu C Long A Fang X Wood SL Slater DM Ni X Olson DM. Effects of PGF2α on the expression of uterine activation proteins in pregnant human myometrial cells from upper and lower segment. J Clin Endocrinol Metab 2013;98:2975–83. 23678036 10.1210/jc.2012-2829

[157] Yang L Cao Z Yu B Chai C. An in vivo mouse model of primary dysmenorrhea. Exp Anim 2015;64:295–303. 25912320 10.1538/expanim.14-0111 PMC4548002

[158] Young M Mikes BA. Uterine fibroid embolization. Treasure Island: StatPearls, 2025. 30085558

[159] Yuan C Liu L Zhao Y Wang K. Puerarin inhibits staphylococcus aureus-induced endometritis through attenuating inflammation and ferroptosis via regulating the P2X7/NLRP3 signalling pathway. J Cell Mol Med 2024;28:e18550. 39042561 10.1111/jcmm.18550 PMC11265464

[160] Yusuf H Trent M. Management of pelvic inflammatory disease in clinical practice. Ther Clin Risk Manage 2023;19:183–92. 10.2147/TCRM.S350750 PMC9939802 36814428

[161] Zhai J Vannuccini S Petraglia F Giudice LC. Adenomyosis: mechanisms and pathogenesis. Semin Reprod Med 2020;38:129–43. 33032339 10.1055/s-0040-1716687 PMC7932680

[162] Zhang H Wu ZM Yang YP Shaukat A Yang J Guo YF Zhang T Zhu XY Qiu JX Deng GZ Shi DM. Catalpol ameliorates LPS-Induced endometritis by inhibiting inflammation and TLR4/NF-kappaB signaling. J Zhejiang Univ Sci B 2019;20:816–27. 31489801 10.1631/jzus.B1900071 PMC6751487

[163] Zhou S Yi T Liu R Bian C Qi X He X Wang K Li J Zhao X Huang C Wei Y. Proteomics identification of annexin A2 as a key mediator in the metastasis and proangiogenesis of endometrial cells in human adenomyosis. Mol Cell Proteomics 2012;11:M112.017988-1–M112.017988-24. 10.1074/mcp.M112.017988 PMC3394960 22493182

[164] Zhu B Zhang C Shen X Chen C Chen X Lu Y Chen Y Guo M. Protective effects of resveratrol against adenomyosis in a mouse model. Dose Response 2023;21:15593258231164055. 36959835 10.1177/15593258231164055 PMC10028632

